# Commonalities and differences in gene expression patterns in major depressive disorder and chronic spontaneous urticaria: implications for comorbidity

**DOI:** 10.3389/fgene.2025.1560832

**Published:** 2025-07-29

**Authors:** Yibo Jiang, Wen Li, Lihao Zheng, Nan Ba

**Affiliations:** The Fifth Affiliated Hospital of Zhengzhou University, Zhengzhou, Henan, China

**Keywords:** chronic spontaneous urticaria (CSU), major depressive disorder (MDD), transcriptomic profiling, sex stratification, comorbidity mechanisms, precision medicine

## Abstract

Chronic Spontaneous Urticaria (CSU) disrupts patients’ physical wellbeing through recurrent wheals that last for up to 24 h and pruritus, and may also contribute to the development and progression of mental health conditions such as depression due to poor control of CSU symptoms. Clinical evidence has shown significant comorbidity between CSU and major depressive disorder (MDD), but our understanding of the underlying molecular mechanisms and how they differ by sex remains limited. In this work, five gene expression datasets were obtained from the Gene Expression Omnibus (GEO), encompassing three for MDD (GSE52790, GSE38206, GSE76826) and two exclusively whole-blood datasets for CSU (GSE167882, GSE72541). Following data preprocessing and batch effect correction, differentially expressed genes (DEGs) in CSU and MDD were identified in male, female, and total populations. Gene Ontology (GO), Kyoto Encyclopedia of Genes and Genomes (KEGG) enrichment analyses were used to characterize putative pathophysiological mechanisms. Subsequently, machine learning algorithms were implemented via 10-fold cross-validation to build transcriptomic classifiers for MDD in a sex-stratified manner. Results indicated shared molecular patterns between CSU and MDD, with 26 key genes in the total population, 6 in males and 7 in females. Further feature selection yielded 6 core transcriptomic features in the total population (*BCL11A, BEX2, C5AR1, DDX60L, LCE3D, NAMPT*), 2 in males (*GNAQ, RNF19B*), and 4 in females (*GNG7, LCE3D, PYGL, UPP1*). Validation analyses showed that these transcriptomic classifiers conferred high classification accuracy, with the model for females surpassing that for males. Potential drug candidates targeting shared molecular mechanisms were identified. Overall, these findings reveal sex-specific molecular signatures that may underlie CSU-MDD comorbidity and offer insight into future precision treatment paradigms.

## 1 Introduction

Chronic Spontaneous Urticaria (CSU) is a common skin disorder characterized by the recurrent appearance of wheals and pruritus, which may vary in intensity and typically lasts for up to 24 h (Zuberbier et al., 2018). The pruritus can recur daily or almost daily and persist for several weeks to months or longer. Global estimates suggest that CSU affects approximately 1%–4% of the population, and its prevalence is higher among young to middle-aged women (Zuberbier et al., 2018). Although CSU is frequently regarded as a dermatological condition, emerging evidence indicates it represents a systemic inflammatory disorder that can impose far-reaching effects extending beyond the physical discomfort of urticarial lesions. Patients often experience substantial emotional and financial burdens (Maurer et al., 2011; Balp et al., 2018), partly because factors such as disease severity, itch intensity, and the unpredictable nature of CSU interfere with daily activities and disrupt overall wellbeing.

There is increasing recognition that CSU and depression share core clinical features including sleep disturbances, fatigue, and impaired quality of life, with growing epidemiological evidence supporting their comorbidity (Staubach et al., 2011; Balp et al., 2018). A growing body of evidence indicates that about 30% of CSU patients exhibit at least one psychiatric comorbidity, which may include depression, anxiety, or somatoform disorders. Depression, in particular, has drawn considerable attention as it correlates with worsened quality of life and can be challenging to treat effectively when superimposed on a chronic inflammatory state (Balp et al., 2018; Kessler and Bromet, 2013; Otte et al., 2016). Some researchers propose that CSU may contribute to the development of depression by fostering chronic stress, heightened immune activation, persistent inflammatory signaling, and sleep impairment caused by itching (Dantzer et al., 2008). Chronic stressors, whether psychological or physiological, have been implicated in the etiology of depression, suggesting an important interplay between the immune system and neuroendocrine function (Dantzer, 2018; Dhabhar, 2009; Miller and Raison, 2016).

Understanding how CSU and depression intersect requires an appreciation of CSU’s pathophysiology. The disease is widely viewed as a systemic inflammatory disorder of the skin that involves mast cell activation and histamine release (Maurer et al., 2016; Zuberbier et al., 2018; Maurer et al., 2011). Two primary autoimmune pathways contribute to CSU: type I autoimmunity, in which IgE autoantibodies bind to autoantigens on mast cells, and type IIb autoimmunity, in which IgG or IgM autoantibodies target IgE or its high-affinity receptor. Both pathways ultimately converge on mast cell degranulation, resulting in localized cutaneous edema, erythema, and pruritus (Zuberbier et al., 2018; Maurer et al., 2011). Intriguingly, these same inflammatory mediators, including histamine, can influence central nervous system pathways implicated in mood regulation. Histamine, in addition to its well-known effects in the peripheral immune system, can also affect the central nervous system, which may contribute to both CSU symptoms and mood disorders. Neurotransmitter imbalances, including those involving serotonin and substance P, may further mediate the connection between sustained inflammation and depressive symptoms (Raison et al., 2006).

Sex-specific factors contribute an additional layer of complexity. Depression is more prevalent among women than men across different cultures, with female patients typically exhibiting higher symptom severity or a more relapsing course (Kuehner, 2017). Fluctuations in sex hormones—such as estrogen and progesterone—can impact how the immune system responds to both internal and external stressors (Klein and Flanagan, 2016; Fish, 2008; Bekhbat and Neigh, 2018). Women may show heightened sensitivity to inflammatory signals in CSU, which could influence depression risk. Conversely, men might exhibit distinct immunological and neuroendocrine responses, making it crucial to investigate differences in molecular markers and clinical trajectories (Klein and Flanagan, 2016; Fish, 2008; Bekhbat and Neigh, 2018).

While classical transcriptomic classifier tools in psychiatry rely heavily on clinical scales and patient self-reports, such subjective approaches can hinder early identification of high-risk individuals (Gangwar et al., 2014). Advances in multi-omics research and machine learning techniques now allow the exploration of integrated datasets to identify molecular indicators of psychiatric disorders (Drysdale et al., 2017; Duc et al., 2019; Grzenda et al., 2021). Given the systemic inflammatory nature of CSU, correlating its molecular underpinnings with depressive phenotypes could offer a promising path toward a more objective, data-driven transcriptomic classifier for MDD (Raison et al., 2013). By stratifying data based on sex, it becomes more feasible to develop nuanced models that address these observed disparities (Klein and Flanagan, 2016; Fish, 2008).

The primary aim of the present study is to elucidate how CSU-related molecular features might facilitate the detection and risk assessment of MDD, particularly with respect to sex-specific differences. By analyzing gene expression data from peripheral blood samples, coupled with immune infiltration profiling, this investigation seeks to propose an transcriptomic classification model that not only enhances our understanding of CSU–MDD comorbidity but also paves the way for targeted therapeutic interventions.

## 2 Methods

### 2.1 Data collection

Publicly available transcriptomic datasets for CSU and MDD were retrieved from the Gene Expression Omnibus (GEO) platform (Edgar et al., 2002). Specifically, GSE52790 (10 MDD, 12 control), GSE38206 (18 MDD, 18 control), and GSE76826 (20 MDD, 12 control) constituted the MDD arm of the analysis, while GSE167882 (15 CSU, 6 control) and GSE72541 (20 CSU, 10 control) were used for CSU. To explore the effect of sex on the CSU–MDD relationship, data from GSE52790, GSE38206, and GSE76826 were further stratified into male and female subgroups. Due to the absence of sex metadata in the CSU datasets, our sex-stratified analyzes were performed exclusively in the MDD cohort. Each dataset underwent normalization steps using the GEOquery R package, facilitating robust cross-dataset comparisons. Table 1 provides the datasets used in the study. 1.

**TABLE 1:** Basic information of GEO datasets used in the study.

Id	GSE series	Disease	Source type	Platform	Total samples	Male	Female
1	GSE52790	MDD	Peripheral blood	GPL17976	10 MDD/12 Control	5 MDD/6 Control	5 MDD/6 Control
2	GSE76826	MDD	Leukocytes	GPL17077	20 MDD/12 Control	9 MDD/5 Control	11 MDD/7 Control
3	GSE38206	MDD	PBMC	GPL13607	18 MDD/18 Control	8 MDD/8 Control	10 MDD/10 Control
4	GSE72541	CSU	Whole blood	GPL16699	20 CSU/10 Control	—	—
5	GSE167882	CSU	Whole blood	GPL23159	15 CSU/6 Control	—	—

CSU, chronic spontaneous urticaria; MDD, major depressive disorder; PBMC, peripheral blood mononuclear cell.

### 2.2 Batch effect removal

Differences in experimental conditions, laboratories, and data acquisition platforms often introduce batch effects that can obscure genuine biological signals. After consolidating the CSU and MDD datasets into male, female, and total population categories, the “ComBat” function from the “sva” R package was employed to correct for batch effects (Leek et al., 2012). Principal Component Analysis (PCA) was performed both before and after correction to ensure that biological variation was retained while technical noise was minimized.

### 2.3 Identification of differentially expressed genes (DEGs)

After normalization and batch correction, each cohort—total population, male, and female—was analyzed to uncover differentially expressed genes (DEGs). The “limma” package was used, applying a threshold of $|\log_{2}\text{FC}|>0.25$ and p<0.05 Illumina (2015). Volcano plots and heatmaps assisted in visualizing the magnitude and significance of gene expression changes. Venn diagrams were constructed to identify genes that were shared between CSU and MDD. To contextualize how these shared DEGs might interact at the protein level, protein–protein interaction (PPI) networks were created using GeneMANIA (http://genemania.org/) (Barbie et al., 2009).

### 2.4 Enrichment analysis of genes shared by MDD and CSU

Functional annotations of the overlapping DEGs in each subgroup—male, female, and total population—were carried out through GO (Gene Ontology) and KEGG (Kyoto Encyclopedia of Genes and Genomes) pathway analyses. R packages including “org.Hs.e.g.,.db,” “ggplot2,” “clusterProfiler,” and “enrichplot” facilitated the enrichment analyses (Barbie et al., 2009). Results were filtered with a significance threshold of p<0.05 to limit false positives and highlight pathways most relevant to immune and neuroendocrine functions implicated in CSU and MDD.

### 2.5 Immune cell infiltration analysis

Single-sample gene set enrichment analysis (ssGSEA) was applied to quantify the levels of 23 immune cell types, ranging from T-cell subsets (CD4^+^, CD8^+^, T helper cells) to dendritic cells and natural killer cells (Cai et al., 2022). Variation in these cell populations was compared across CSU and MDD samples versus controls. Special emphasis was placed on determining how these patterns might differ between men and women, given the known influence of sex hormones on immune regulation. This analysis provided insights into whether certain cell types, such as mast cells or pro-inflammatory Th1 cells, were enriched in specific cohorts.

### 2.6 Machine learning algorithms to construct a transcriptomic classifier for depression

Machine learning algorithms were employed to construct transcriptomic classifiers for depression based on the shared DEGs and immune cell infiltration profiles. Specifically, 12 different machine learning algorithms, including Elastic Net (Friedman et al., 2010), randomForest (Liaw and Wiener, 2002), and XGBoost (Chen and Guestrin, 2016), were utilized. Each model scenario was evaluated using 10-fold cross-validation to ensure robustness and generalizability.

### 2.7 Identification of drug candidates

To explore potential therapeutic implications, genes central to each final model were evaluated using the Drug Signature Database (DSigDB) on the Enrichr platform (https://amp.pharm.mssm.edu/enrichr/) (Kuleshov et al., 2016). This step aimed to pinpoint approved drugs or experimental compounds that might modulate the molecular pathways overlapping CSU and MDD, offering translational opportunities for future research (Keiser et al., 2007; Trott and Olson, 2010).

### 2.8 Statistical analyses

All statistical processes were handled in R (version 4.2.2). The limma package was used for DEG identification, with p<0.05 deemed significant. When comparing transcriptomic classifiers, the DeLong test was employed to determine whether differences in AUC values were statistically meaningful (Dhabhar, 2009).

## 3 Results

### 3.1 Data processing

**FIGURE 1:**
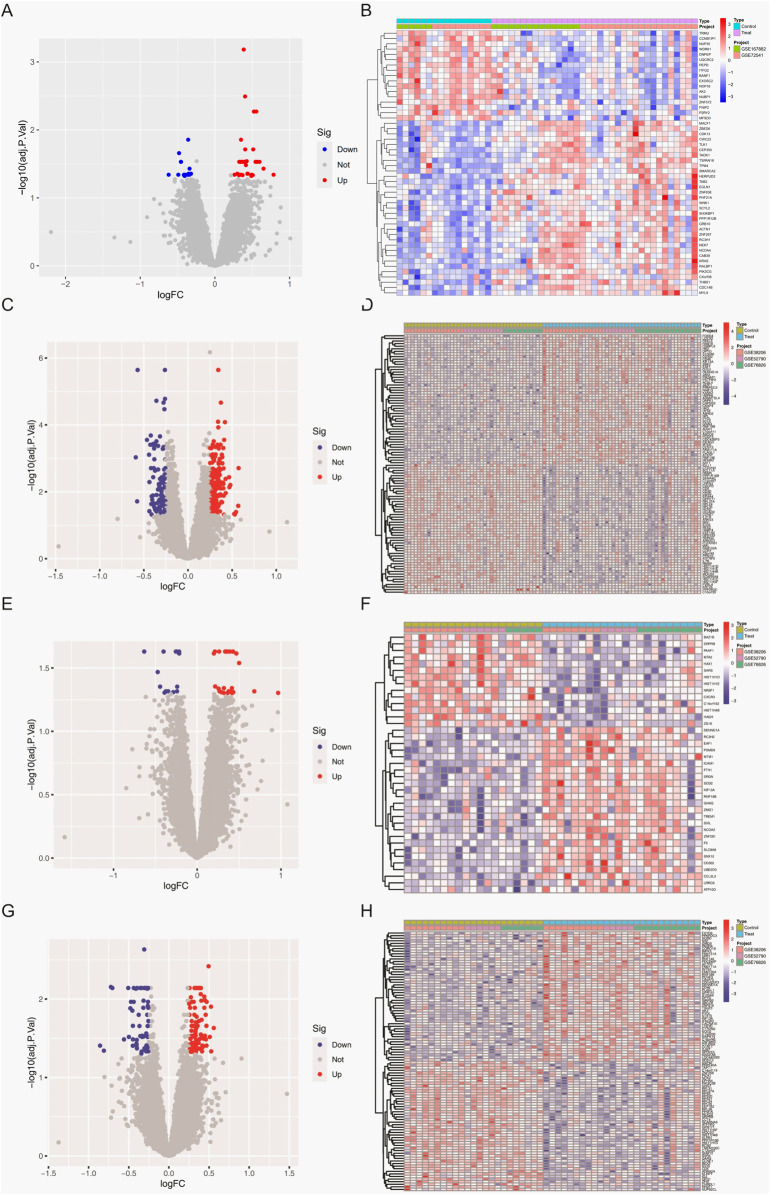
Differentially expressed genes (DEGs) in whole-blood samples. **(A)** Volcano plot depicting DEGs between CSU patients and healthy controls. **(B)** Heatmap showing DEGs between CSU patients and healthy controls. **(C)** Volcano plot depicting DEGs between the total MDD population and healthy controls. **(D)** Heatmap showing DEGs between the total MDD population and healthy controls. **(E)** Volcano plot depicting DEGs between male MDD patients and healthy controls. **(F)** Heatmap showing DEGs between male MDD patients and healthy controls. **(G)** Volcano plot depicting DEGs between female MDD patients and healthy controls. **(H)** Heatmap showing DEGs between female MDD patients and healthy controls.

Figure 1 provides the expression profile of all genes. Raw transcriptomic data from GSE167882 and GSE72541 were combined for the CSU cohort (35 CSU, 16 controls), while GSE52790, GSE38206, and GSE76826 were merged to form an MDD cohort (48 MDD, 42 controls). Sex stratification of MDD data further separated the samples into 22 MDD and 19 controls (male), and 26 MDD and 23 controls (female). Through PCA, we confirmed that the “ComBat” batch correction effectively mitigated spurious inter-dataset variation, enabling consistent comparisons across different platforms and experimental conditions.

### 3.2 Results of differentially expressed gene analysis

**FIGURE 2:**
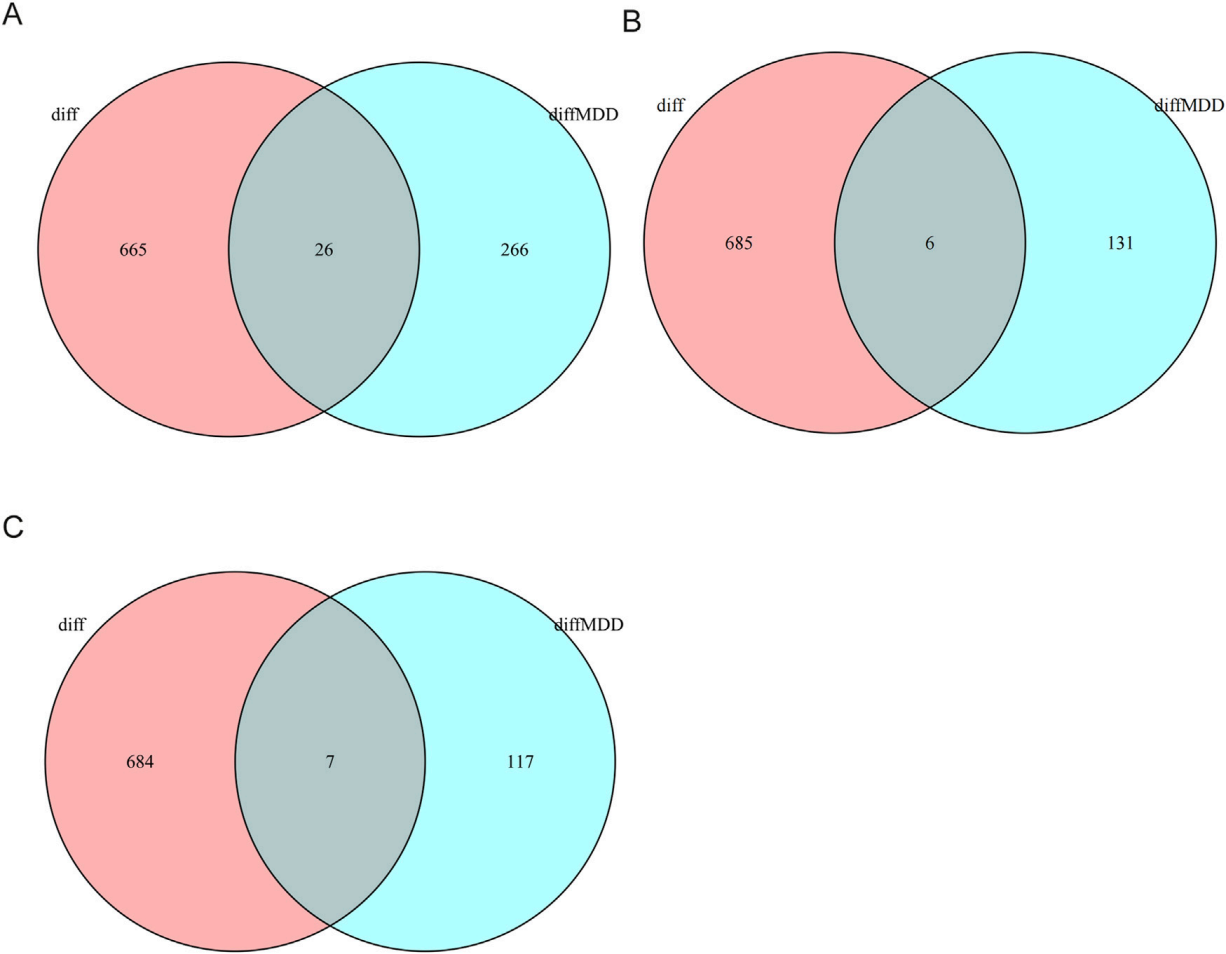
Venn diagrams indicating DEGs shared between CSU (whole-blood) and MDD (whole-blood/PBMC). **(A)** Venn diagram showing shared DEGs between CSU and the total MDD population. **(B)** Venn diagram showing shared DEGs between CSU and male MDD patients. **(C)** Venn diagram showing shared DEGs between CSU and female MDD patients.

An initial screening of the CSU data sets identified 690 differentially expressed genes (DEGs) according to the criteria of $|\log_{2}\text{FC}|>0.25$ and FDR <0.05 (Supplementary Figure S1A, B) Demkow and Wolańczyk (2017). Analysis of the MDD datasets in the total population yielded 285 DEGs, whereas separate evaluations of the male and female subgroups recovered 39 and 161 DEGs, respectively (Supplementary Figure S1C–H). To explore potential sex-specific overlaps between CSU and MDD pathophysiology, we compared CSU-related DEGs (analyzed in the total population due to lack of sex metadata) with MDD-related DEGs stratified by sex. This intersection revealed 26 shared genes in the overall cohort. Notably, when CSU-associated DEGs were compared against male- and female-specific MDD DEGs, 2 overlapping genes were identified in males and 4 in females. (Figures 2A–C). Many of these overlapping genes were implicated in pathways pertaining to immune regulation and neuroinflammation. Supplementary Material.

### 3.3 Functional annotation and enrichment analysis of core genes

**FIGURE 3:**
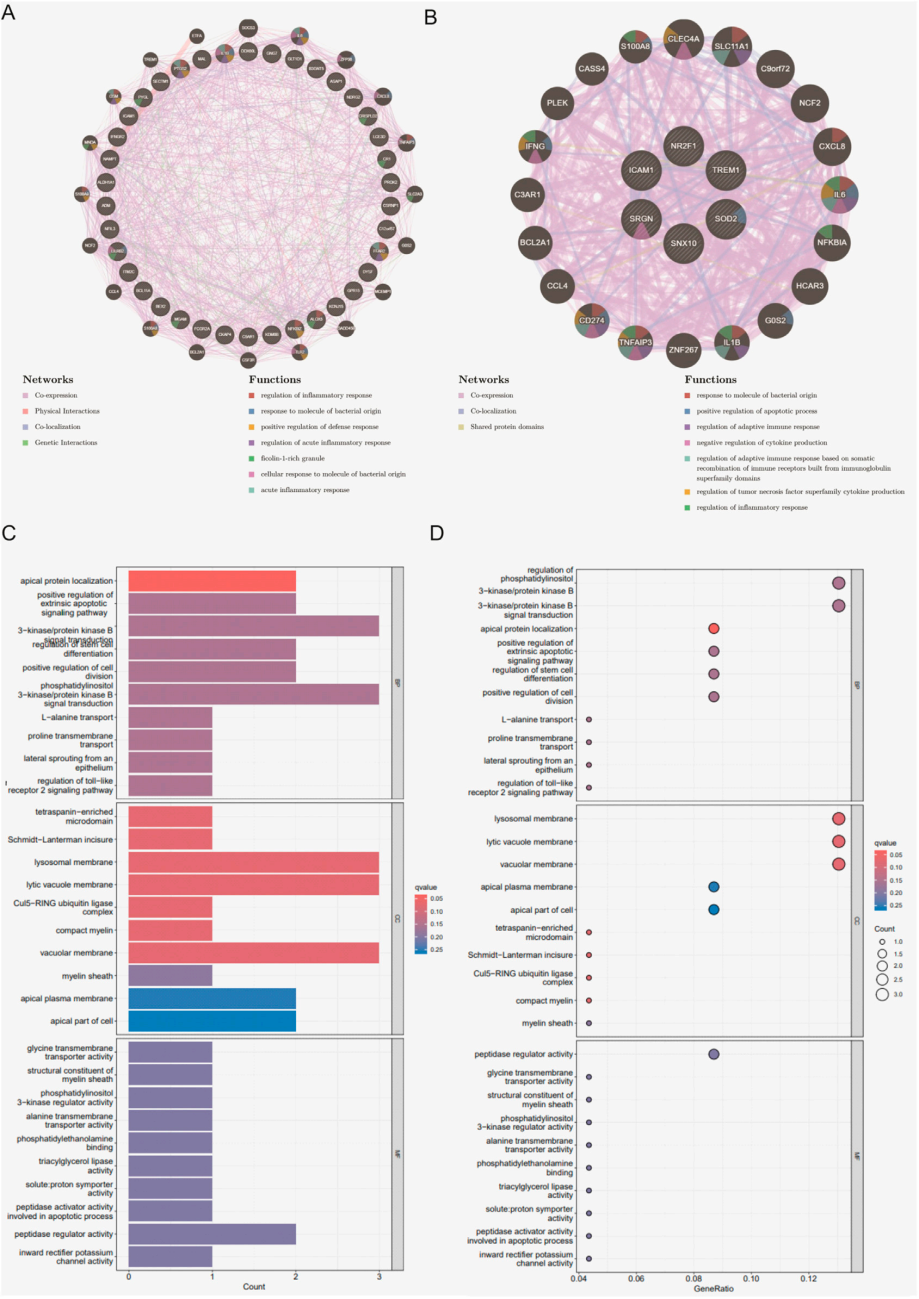
PPI network analysis and enrichment analyses for shared DEGs across total, male, and female groups. **(A)** PPI network of 26 shared DEGs in the total population constructed by GeneMANIA. **(B)** PPI network of 6 shared DEGs in the male group. **(C)** Bar plot of GO enrichment analysis results in the CSU and MDD total population. **(D)**Bubble plot of GO enrichment analysis results in the CSU and MDD total population.

To uncover the potential biological roles of the shared DEGs, PPI networks were built with GeneMANIA, followed by GO and KEGG enrichment analyses. In the total population, these genes were primarily associated with the regulation of inflammatory and immune defense functions. In males, the DEGs leaned toward cytokine signaling and acute immune responses, while those in females showed a clear inclination toward inflammatory pathways intersecting with neuroendocrine regulation (Figures 3A,B). GO analysis indicated that genes in the overall group were enriched in T-cell-mediated immunity and vascular endothelial growth factor production, among other processes. The male subgroup’s DEGs were largely involved in acute inflammatory or antiviral immune responses, whereas the female subgroup showed enhanced enrichment in angiogenesis, neuroinflammatory signals, and metabolic adjustments (Figures 3C,D; Supplementary Figure S1).

Further insight emerged from KEGG pathway annotations: cytokine–cytokine receptor interactions, Toll-like receptor signaling, and the JAK–STAT pathway featured prominently in the total population. Conversely, females showed enrichment in PI3K–Akt signaling and ECM–receptor interactions, which together underlined complex regulatory changes in cell survival, tissue repair, and metabolic control. Males, however, remained concentrated in pathways that mount rapid inflammatory and viral defense responses (Supplementary Figure S2). These findings collectively pointed to overlapping but diverging mechanisms within CSU and MDD, shaped partly by sex-mediated immune and endocrine processes.

### 3.4 Results of immune cell infiltration analysis

**FIGURE 4:**
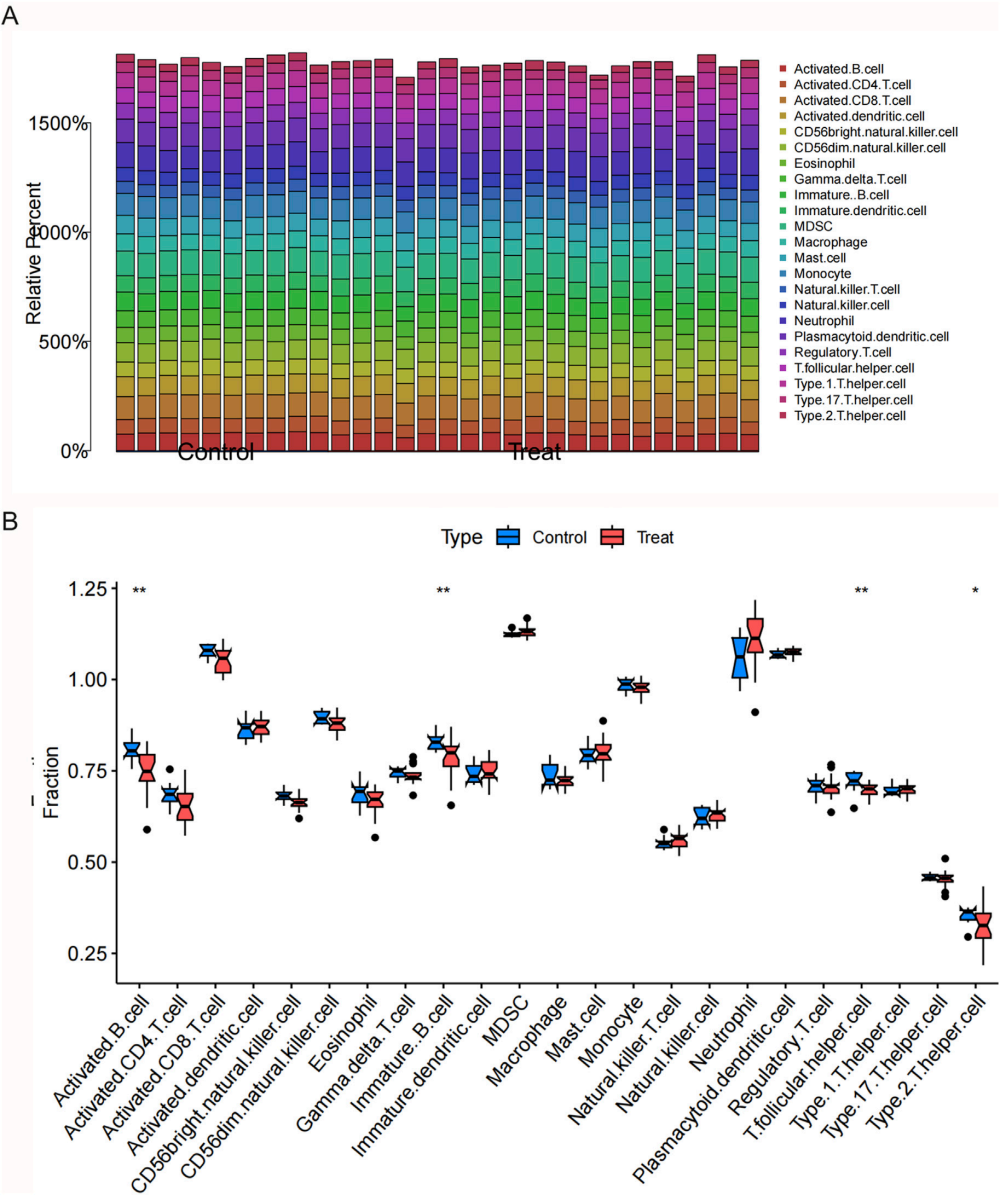
Immunological characteristics in CSU and MDD across total, male, and female groups. **(A)** Bar plot showing immune cell abundances in CSU patients and controls (total population). **(B)** Immune cell analysis (boxplots) comparing immune cell abundances in CSU patients and controls (total population).

Results from the ssGSEA-based immune cell infiltration analysis revealed that multiple immune cell subsets were differentially represented in both CSU and MDD samples when compared with controls (Figure 4, Supplementary Figure S3). In the total population, monocytes, dendritic cells (both immature and plasma-like), and activated CD4^+^ T cells appeared elevated in CSU, and a similar pattern emerged in MDD, suggesting a persistent state of immune activation. Stratifying by sex unveiled more nuanced differences. In MDD females exhibited a marked increase in pro-inflammatory cell types, such as neutrophils and Th1 helper T cells, alongside heightened mast cell activity. This set of findings aligns with clinical observations that some women may exhibit an exacerbated immune response, possibly tied to hormonal influences or stress reactivity. In MDD male samples, on the other hand, reflected notable upregulation in regulatory T and B cells, hinting that men might compensate for immune challenges in distinct ways (Supplementary Figures S4, S5).

These sex-specific transcriptomic patterns not only underscore the complexity of CSU and MDD comorbidity but also raise intriguing possibilities for tailored treatment. For example, immunomodulatory therapies that directly target regulatory T-cell or B-cell function could be explored in comorbid CSU-MDD patients.

### 3.5 Results of machine learning model evaluation

**FIGURE 5:**
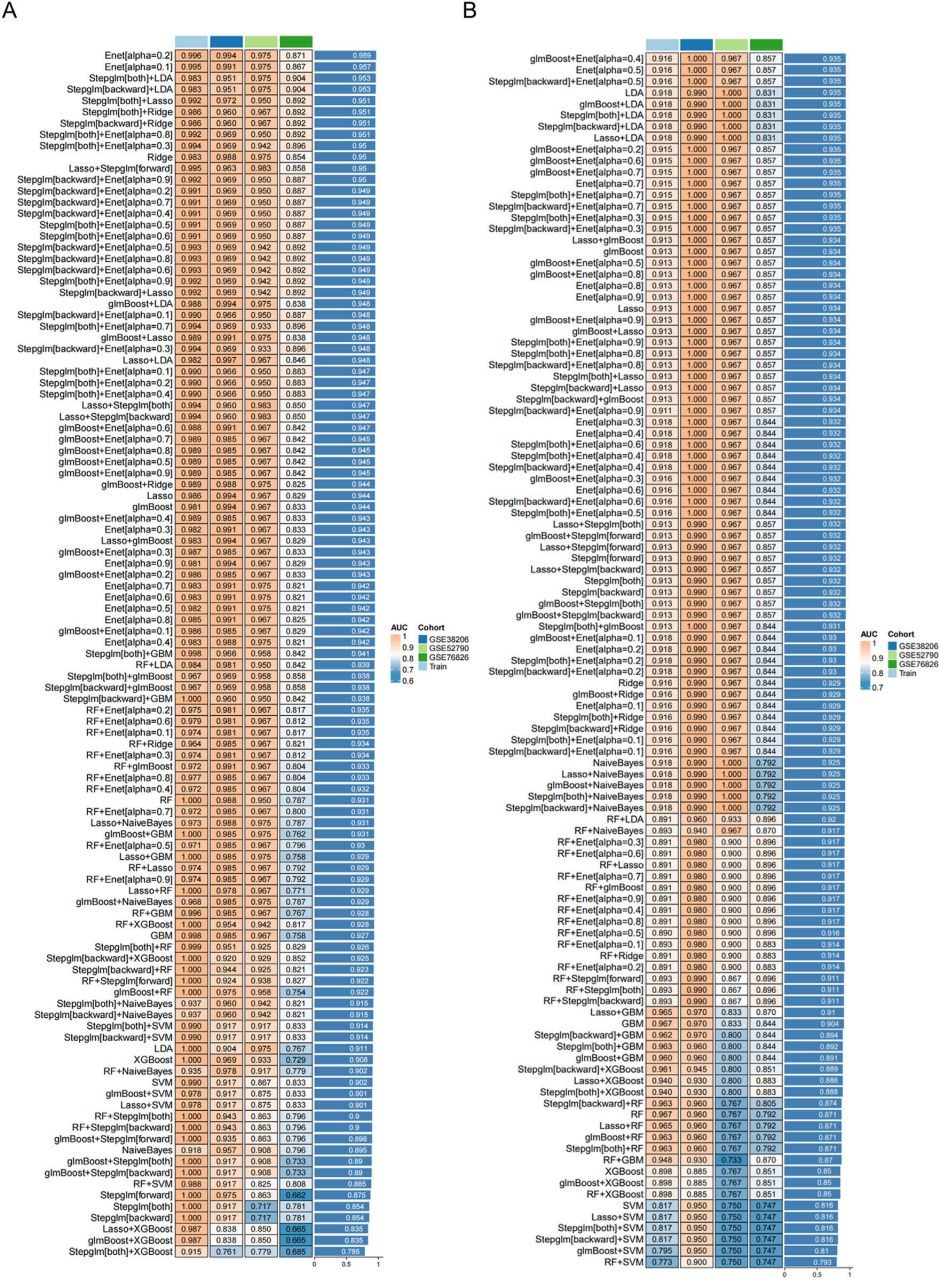
Receiver operating characteristic (ROC) curves of transcriptomic classifier performance for distinguishing MDD patients from healthy controls using whole-blood gene expression. Models trained on: Total population: MDD (GSE52790/GSE38206/GSE76826) vs. controls Sex subgroups: Stratified MDD cohorts vs. controls: **(A)** Machine learning transcriptomic classifier evaluated for the total population. **(B)** Machine learning transcriptomic classifier evaluated for the female group.

Building on the shared DEGs and immune insights, 113 machine learning model scenarios were constructed and tested. Ten-fold cross-validation helped balance bias and variance, ensuring that the final models were both robust and generalizable. The optimal approach integrated a backward feature selection (Stepglm [backward]) with the Enet [alpha = 0.1] (α=0.1) , ultimately extracting 6 key genes in the total population (BCL11A, BEX2, C5AR1, DDX60L, LCE3D, NAMPT), 2 in males (GNAQ, RNF19B), and 4 in females (GNG7, LCE3D, PYGL, UPP1). (Liaw and Wiener, 2002; Chen and Guestrin, 2016) (Figure 5, Supplementary Figure S6). These signature genes encompass factors involved in immune regulation, neuroinflammation, and metabolic processes—reinforcing the suspicion that both immunological and neurochemical pathways may be implicated in CSU–MDD comorbidities.

Subsequent evaluations, including ROC curves and calibration assessments, demonstrated strong classification capability. The total population model achieved an AUC of around 0.905, while the female subgroup notably reached approximately 0.945, surpassing the ∼0.911 observed in males Supplementary Figures S7-S9). Statistical comparisons with previously published transcriptomic classifiers further verified the superior performance of the CSU-based framework. The identification of these core genes has research relevance for understanding shared molecular mechanisms. Future studies in cohorts of CSU patients with/without MDD are needed to validate their predictive value for depression risk. But these models do not predict the risk of MDD in CSU patients due to the lack of comorbid cohort data.

### 3.6 Comparative analysis with existing models

**FIGURE 6:**
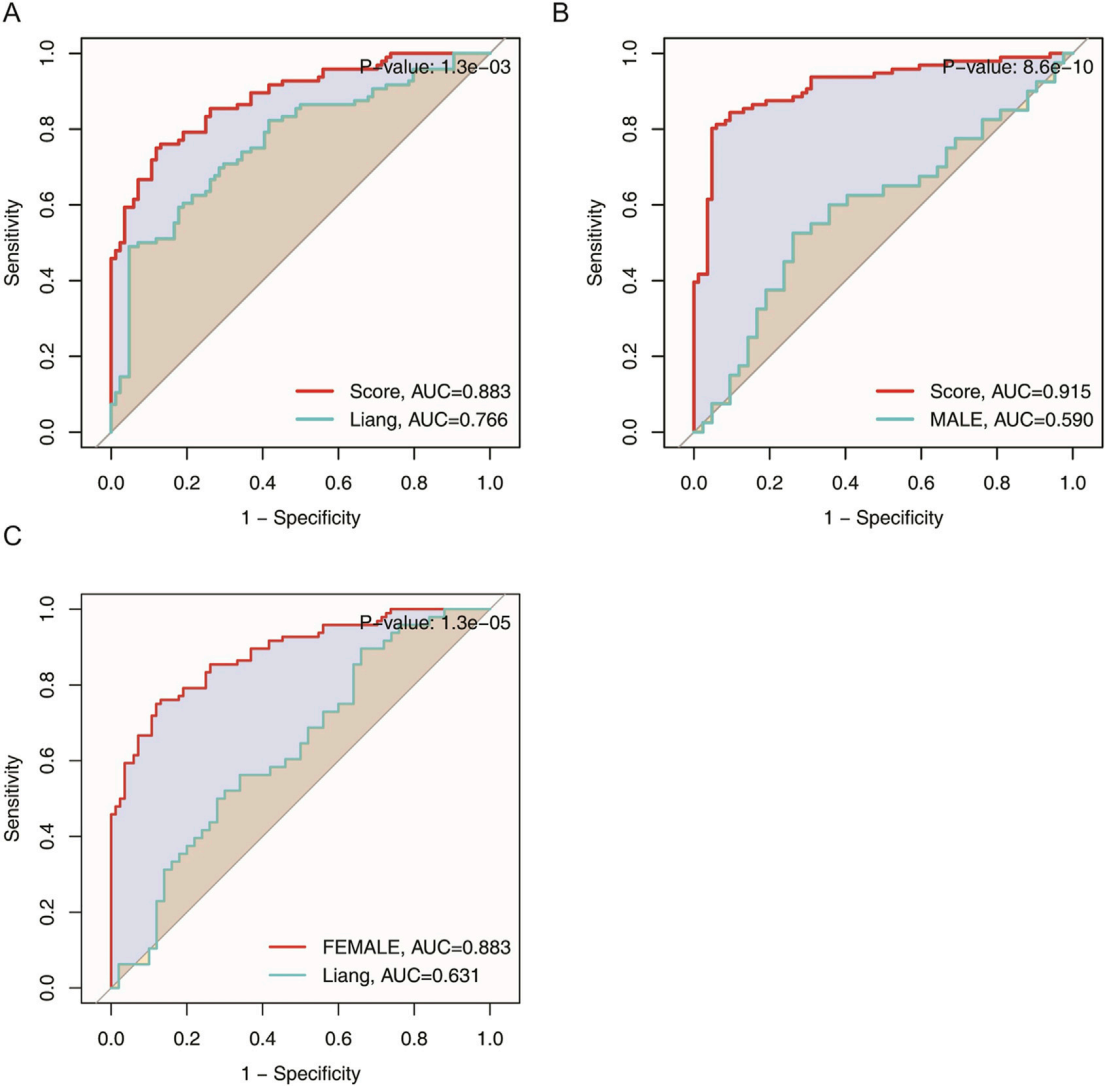
Area under the curve (AUC) comparison of transcriptomic classifier gene expression signatures for MDD across the total population, male, and female groups. **(A)** ROC curve comparing the current model to Liang et al.‘s model in the total population. **(B)** ROC curve comparing the current model to Liang et al.‘s model in the male group. **(C)** ROC curve comparing the current model to Liang et al.‘s model in the female group.

When placed against transcriptomic classifier tools developed purely from inflammatory or aging-related markers, such as approach, the present model consistently yielded higher AUCs, a difference confirmed to be statistically significant via the DeLong test (p<0.05) (Figure 6). This underscores that incorporating disease-specific immunological features—here, derived from CSU—can enhance detection of depression. Rather than relying on generic markers, the synergy between CSU and MDD at the molecular level may provide a more focused and discriminating perspective. These comparisons highlight the importance of cross-domain integration in constructing predictive models, ensuring greater translational potential and clinical utility.

### 3.7 Results of drug candidate screening

Insights into possible therapeutic interventions arose from screening the final model genes against the DSigDB database on Enrichr (Kuleshov et al., 2016). Several promising drug candidates emerged, differentiated according to the total population and by sex. In females, where pro-inflammatory signaling and mast cell activity appeared enhanced, anti-inflammatory agents combined with selective serotonin reuptake inhibitors (SSRIs) were identified. This dual-target strategy could reduce cutaneous inflammatory markers and modulate neurotransmitter imbalances often seen in MDD. Meanwhile, male-centric results emphasized IL-6 signaling pathway inhibitors and HPA-axis modulators, implying that adjusting the balance of cytokines and neuroendocrine factors might be particularly valuable. Although IL-6 inhibitors show promise for modulating inflammatory pathways shared by CSU and MDD, current evidence does not support their routine clinical use in CSU patients and warrants further investigation in comorbid populations. In the aggregated total population, immunomodulatory and anti-inflammatory compounds again took center stage, reflecting the overall importance of dampening chronic inflammation to alleviate both dermatologic and psychiatric burdens Tables 2–4 (Keiser et al., 2007; Trott and Olson, 2010).

**TABLE 2:** Gene-targeted drugs for MDD and CSU in the total population group.

Name	P-value	Adjusted P-value	Odds ratio	Combined score
bromocriptine HL60 UP	0.001228	0.2187	45.82	307.11
progesterone CTD 00006624	0.005341	0.2187	5.92	30.95
cytarabine CTD 00005743	0.005968	0.2187	9.85	50.42
diazoxide CTD 00005792	0.009064	0.2187	128.04	602.21
Fonofos CTD 00005884	0.009837	0.2187	8.16	37.70
TERBUFOS CTD 0000658	0.009951	0.2187	8.12	37.44
parathion CTD 00006472	0.01243	0.2187	7.46	32.72
Rosuvastatin CTD 00003899	0.01293	0.2187	87.58	380.84
cytochalasin B MCF7 UP	0.01485	0.2187	75.63	318.35
SELENIUM CTD 00006731	0.01486	0.2187	5.27	22.18

**TABLE 3:** Gene-targeted drugs for MDD and CSU in the male group.

Name	P-value	Adjusted P-value	Odds ratio	Combined score
L-sorbose CTD 00006006	0.00008268	0.004200	907.91	10,625.40
dexamethasone CTD 00005779	0.00003647	0.009263	140.51	1435.91
T0901317 CTD 00003912	0.00005835	0.009881	326.80	3186.01
dihydroergotamine HL60 UP	0.0002730	0.03466	148.22	1216.36
Pyrrolidine dithiocarbamate CTD 00001021	0.0003729	0.03479	126.36	997.53
ciclopirox HL60 UP	0.0004619	0.03479	113.26	869.89
ciglitazone CTD 00001835	0.0005319	0.03479	105.36	794.32
progesterone BOSS	0.0007187	0.03479	90.31	653.64
[6-[6-(butanoylamino)…]	0.0007383	0.03479	89.07	642.32
vinblastine CTD 00006986	0.0007581	0.03479	87.87	631.33

**TABLE 4:** Gene-targeted drugs for MDD and CSU in the female group.

Name	P-value	Adjusted P-value	Odds ratio	Combined score
letrozole HL60 UP	0.007477	0.09489	166.42	814.75
flunisolide HL60 UP	0.007776	0.09489	159.75	775.88
halcinonide HL60 UP	0.008074	0.09489	153.60	740.22
fludroxycortide HL60 UP	0.008372	0.09489	147.90	707.41
flumetasone HL60 UP	0.008372	0.09489	147.90	707.41
Benzamide CTD 00001769	0.008372	0.09489	147.90	707.41
diflorasone HL60 UP	0.008670	0.09489	142.61	677.12
lithocholic acid HL60 UP	0.009563	0.09489	128.79	598.87
CADMIUM SELENIDE CTD 00002452	0.009860	0.09489	124.76	576.31
alclometasone HL60 UP	0.01016	0.09489	120.98	555.22

## 4 Discussion

Major Depressive Disorder (MDD) persists as a leading cause of disability worldwide, with an estimated 3.8% prevalence that is poised to increase in the coming decades (Kessler and Bromet, 2013). Clinical transcriptomic classifiers are based on subjective indicators, which poses a serious challenge to health systems looking for early detection and cost-effective management (Demkow and Wolańczyk, 2017). Clinical transcriptomic classifiersis combined with molecular biomarkers can supplement clinical transcriptomic classifiersis in the future. The current study addresses these difficulties by integrating bioinformatics, immunological profiling, and machine learning, all within the framework of a systemic inflammatory disorder like CSU, which involves the immune system both locally and systemically. The rationale for choosing CSU is anchored in its established immune-mediated pathophysiology, which may mirror or amplify the inflammatory components of depression, and in severe cases with poor symptom control, CSU may contribute to the development of depression (Maurer et al., 2011).

The shared gene expression patterns between CSU and MDD raise intriguing mechanistic questions about the interplay of chronic inflammation, mast cell degranulation, and alterations in central neurotransmitters. Observations from the present analyses suggest that immune dysregulation—specifically, heightened monocyte and dendritic cell activity, as well as T-cell imbalance—could converge on pathways that regulate mood and emotional states. Substance P, neurotensin, and other neuropeptides known to be released upon mast cell activation can cross-influence stress responses, potentially linking the pathophysiology of CSU and depressive symptomatology (Raison et al., 2006; Dantzer, 2018). The notion that sex hormones further modulate these circuits provides a compelling argument for analyzing data along sex lines, a point substantiated by the different distributions of immune cells and distinct sets of DEGs identified in males and females.

Further strengthening the case for integrative analysis, the shared DEGs identified in this study (e.g., BCL11A, BEX2, C5AR1, DDX60L, NAMPT) converge on key neuroimmune-metabolic pathways relevant to CSU-MDD comorbidity. BCL11A functions as a transcriptional regulator that maintains hematopoietic stem cell quiescence by suppressing Fcer1g-mediated Fc receptor signaling, yet paradoxically accelerates inflammatory aging through IL-1-driven microglial priming in chronic stress models. This dual role may explain its dysregulation in both cutaneous inflammation and depression comorbidity (Zhang, 2016). C5AR1 (C5a receptor) amplifies neuroinflammation through complement-dependent glial activation, with recent evidence showing its antagonism suppresses IL-1/IL-6 production and preserves neuronal integrity (Tenner, 2024). NAMPT, the rate-limiting enzyme in NAD + biosynthesis, emerges as a pivotal metabolic-immune integrator. Its deficiency disrupts NAD + -dependent deacetylation of NF-B, unleashing neurotoxic inflammation (Miller and Raison, 2016).

Sex differences among MDD patients were underscored by the superior performance of the female-specific transcriptomic classifier, which reached an AUC of ∼ 0.945. These findings dovetail with epidemiological data that indicate a higher prevalence and greater severity of depression in women, potentially driven by hormone-related fluctuations in immune function (Kuehner, 2017; Albert, 2015; Marcus et al., 2005). Future validation of this transcriptomic classifier requires larger studies focusing on sex-specific mechanisms, which may improve early detection strategies for depression in CSU patients.

A salient feature of this study is its potential translational application. While many advanced machine learning approaches are tested in research settings, only a subset are clinically embraced due to issues of overfitting, lack of validation, or reliance on data that is difficult to measure in routine practice. The chosen approach of peripheral blood gene expression is relatively feasible for large-scale screening, and the cross-validation strategies deployed here minimize bias. Nevertheless, external validation using independent cohorts, as well as prospective trials, would help confirm the robustness and reproducibility of these findings.

This study identifies molecular features linking Chronic Spontaneous Urticaria (CSU) and Major Depressive Disorder (MDD) through shared transcriptomic patterns. We also consider the role of metabolic adjustments associated with mast cell activation, which could influence both inflammation and mood regulation. Changes in energy metabolism and lipid profiles are relevant to both conditions, and further metabolomic research may offer additional insights. The JAK-STAT pathway emerged as a potential therapeutic target, with JAK inhibitors representing a promising approach for concurrent CSU and MDD management. While promising therapeutic candidates were identified, we acknowledge the limitations of drugs like corticosteroids and progesterone due to their side effects and potential to exacerbate urticaria (Gangwar et al., 2014).

The observed enrichment of necroptosis-related genes in female MDD patients may reflect molecular-level interactions relevant to inflammatory comorbidities. As a programmed cell death modality, necroptosis has emerging roles in chronic inflammatory disorders (Pasparakis and Vandenabeele, 2015). Future work should validate these expression patterns in dedicated comorbidity cohorts.

Drug discovery insights gained through Enrichr further underscore the practical opportunities to explore interventions for CSU–MDD comorbidity (Kuleshov et al., 2016; Keiser et al., 2007; Trott and Olson, 2010). For instance, SSRIs have historically been used to manage depressive episodes, but evidence of their anti-inflammatory properties could be harnessed for integrated treatment (Rosenblat and McIntyre, 2018). Meanwhile, IL-6 inhibitors might be repurposed for patients showing strong upregulation of IL-6 signaling (Raison et al., 2013). The potential synergy of immunomodulation and neurotransmitter rebalancing warrants further clinical investigation.

## 5 Limitations

This study integrates multiple datasets from the GEO repository, but several limitations remain. The small sample size, particularly when divided by sex, may limit the detection of subtle genetic or immunological differences. The heterogeneity of sample collection methods, patient backgrounds, and disease severity across studies also introduces variability. The datasets did not include key clinical parameters, such as age, disease duration, symptom severity, sleep impairment, QoL, treatment response, and the use of biological drugs. Including these in future studies would improve our understanding of the CSU–MDD comorbidity. Moreover, functional validation of the identified genes and biomarkers has not been conducted, and their transcriptomic classifier value requires further validation through larger, independent cohorts. The study’s findings should be considered preliminary, with future research needed to confirm and expand upon these results.

## 6 Conclusion

In conclusion, analysis of CSU reveals immune-mediated pathways that may exacerbate depression symptoms in comorbid conditions, highlighting potential targets for treating CSU-MDD comorbidity. These findings offer a framework for targeting shared immunological factors in comorbid CSU-MDD, with implications for both psychodermatology and precision medicine. By combining bioinformatics techniques with immune infiltration assessments and machine learning, we uncovered key gene signatures—some overlapping in men and women, while others are unique to each sex—that can predict depression risk among CSU patients. The identification of distinct pathways in female and male subgroups underscores the importance of sex stratification in psychiatric biomarker research. Moreover, integrative drug candidate screening suggests multiple avenues for targeted therapies that attenuate inflammatory cascades and potentially modulate neuroendocrine processes.

Beyond clarifying how immune dysregulation might unify CSU and MDD, these results lay the groundwork for more precise, personalized medicine approaches, highlighting the role of immune system imbalances in both conditions and suggesting potential therapeutic targets that could address both CSU and depression. Future studies could focus on validating and refining these models in larger, prospectively collected cohorts, as well as expanding investigation into how advanced treatments—such as biologics targeting specific cytokines or combined immuno-psychiatric therapies—might benefit patients with concurrent CSU and depression. Ultimately, the synergy between dermatological and psychiatric research exemplified in this study may serve as a blueprint for tackling other complex conditions driven by systemic inflammation and comorbid mental illness.

## Data Availability

The original contributions presented in the study are included in the article/Supplementary Material, further inquiries can be directed to the corresponding author.
