# Supplementary figures and images for: Commonalities and differences in gene expression patterns in major depressive disorder and chronic spontaneous urticaria: implications for comorbidity
Source: Front Genet. 2025 Jul 29;16:1560832. doi: 10.3389/fgene.2025.1560832 (PMC12339353; doi:10.3389/fgene.2025.1560832)

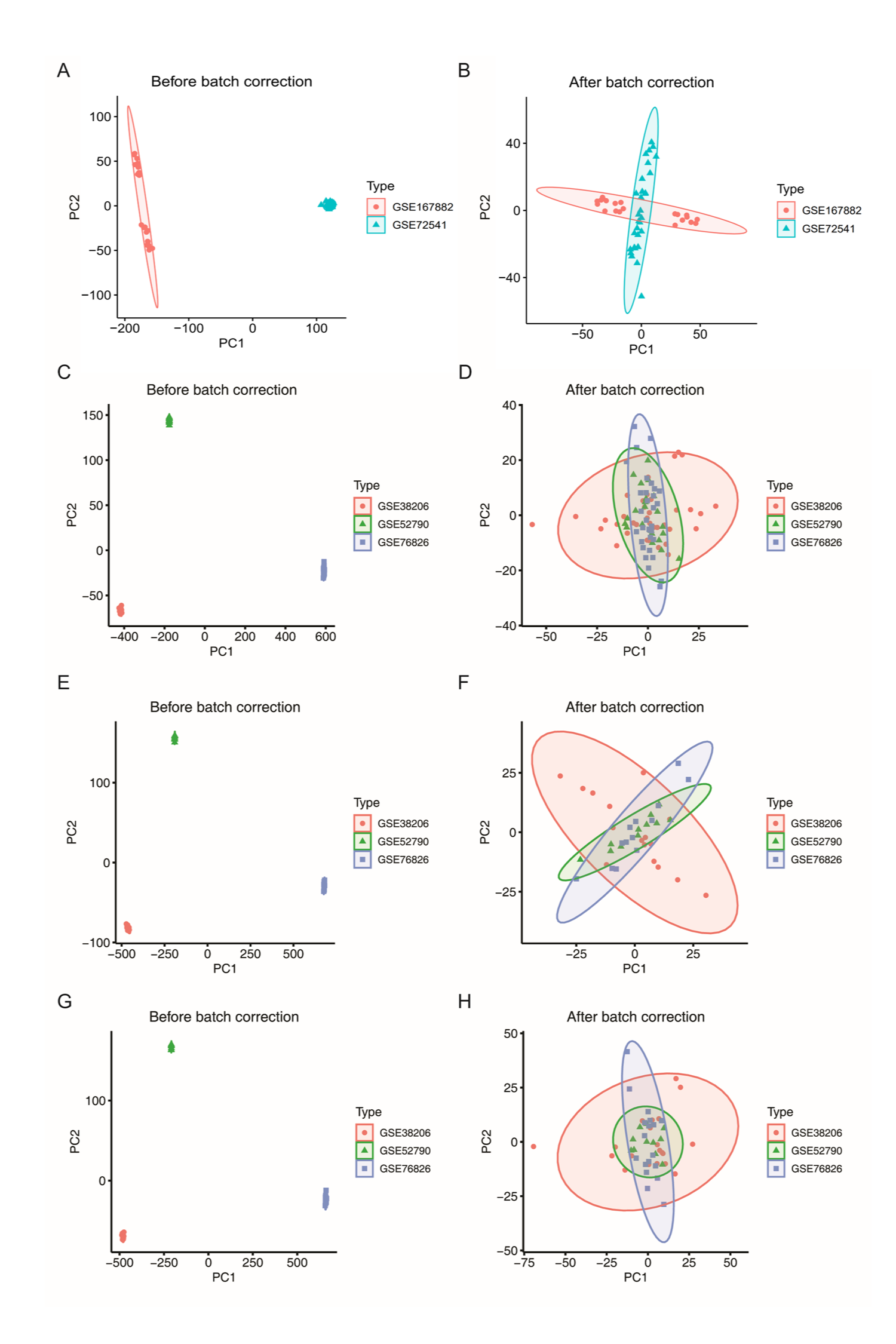

Supplement: Supplementary file 1 [file DataSheet1.zip › S1-S10/fig.S1.TIF]

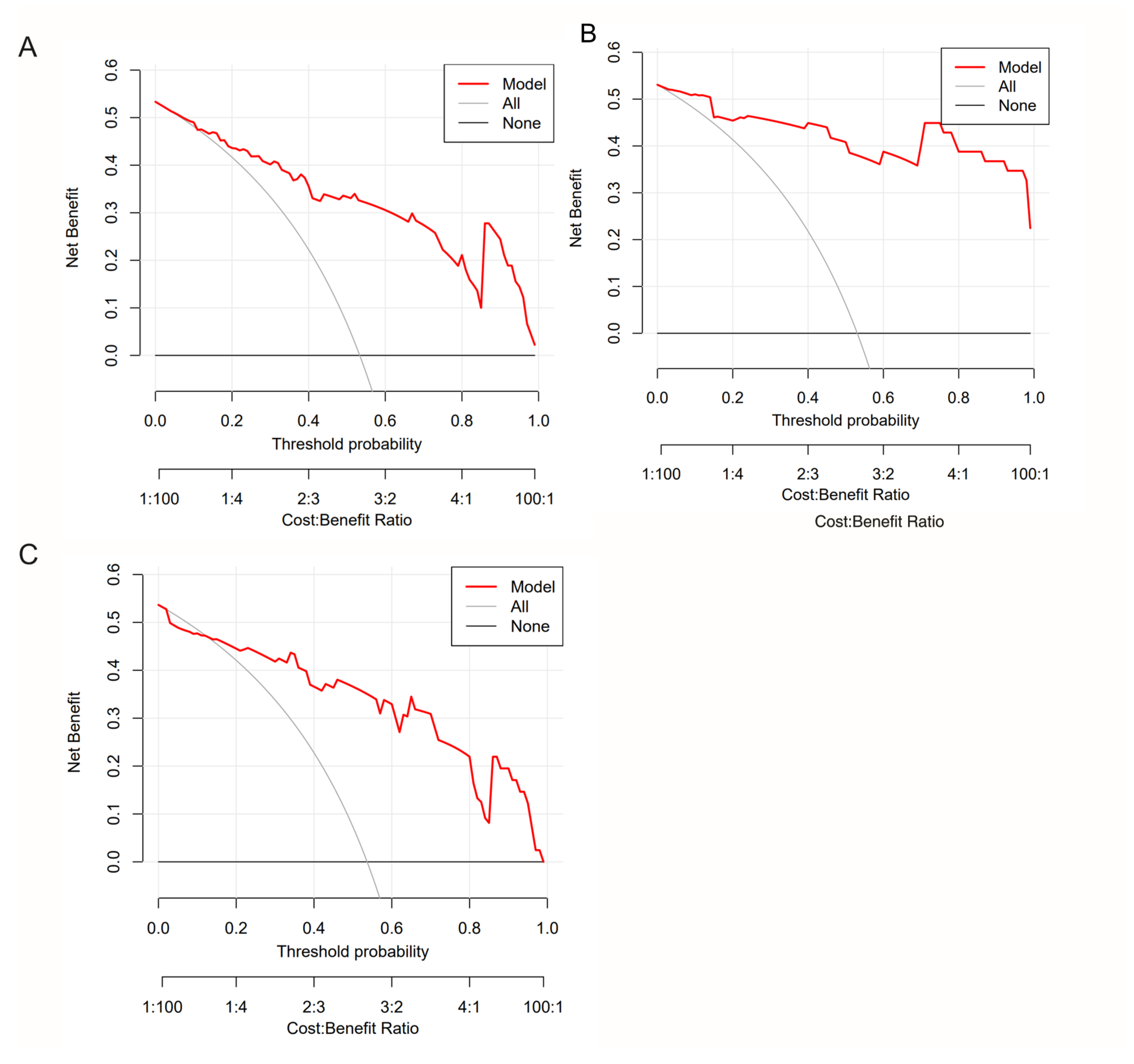

Supplement: Supplementary file 1 [file DataSheet1.zip › S1-S10/fig.S10.TIF]

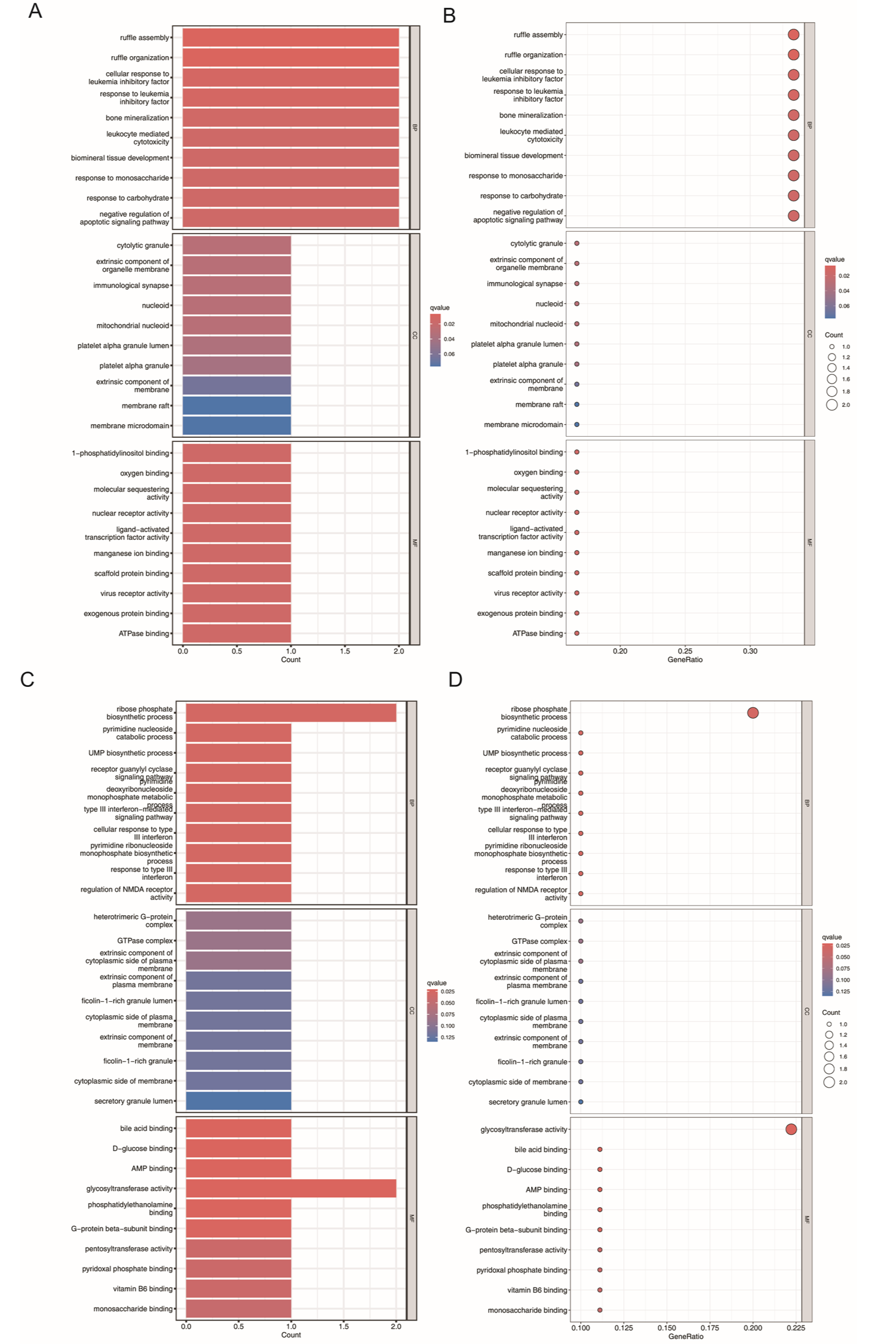

Supplement: Supplementary file 1 [file DataSheet1.zip › S1-S10/fig.S2.TIF]

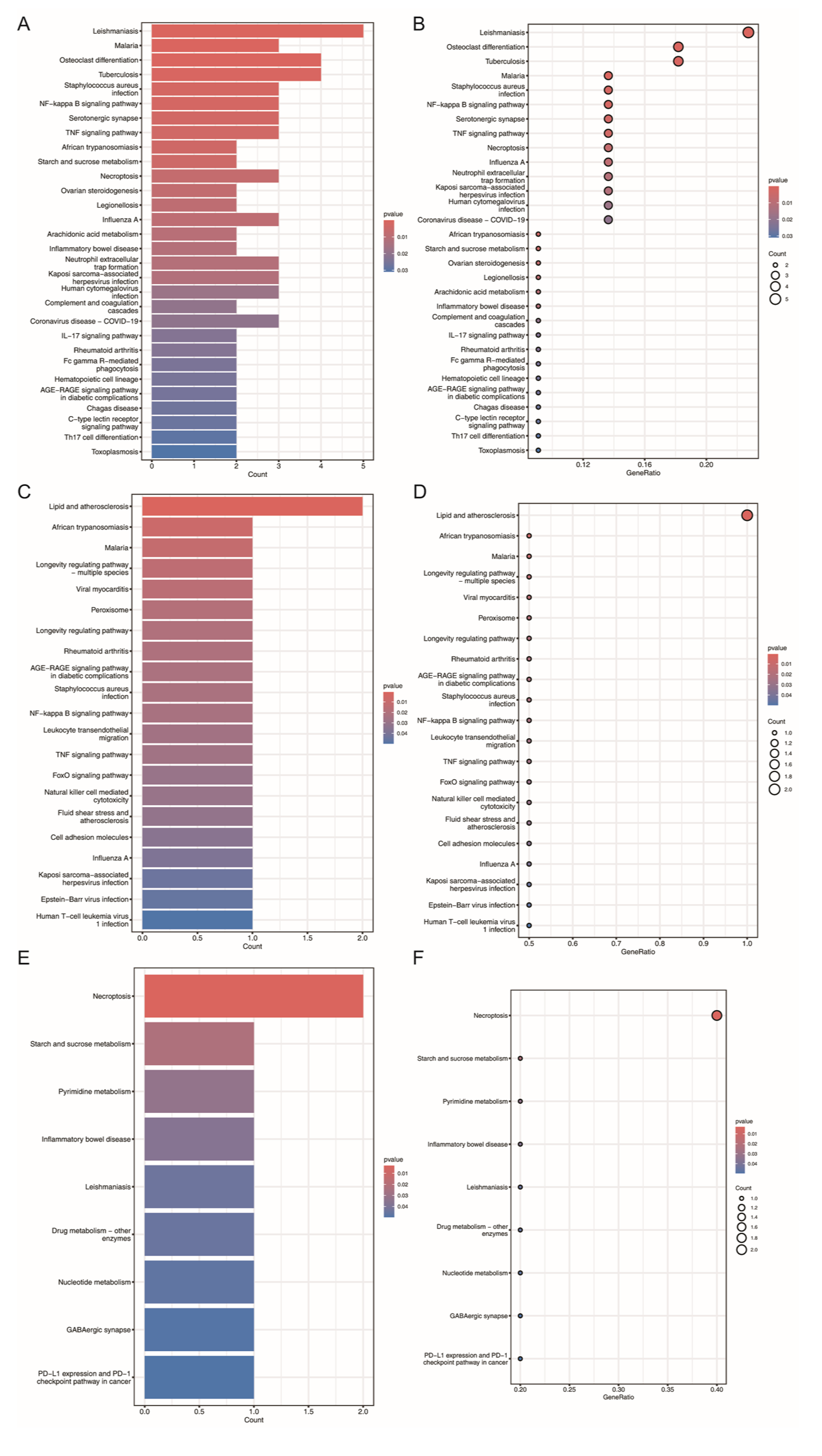

Supplement: Supplementary file 1 [file DataSheet1.zip › S1-S10/fig.S3.TIF]

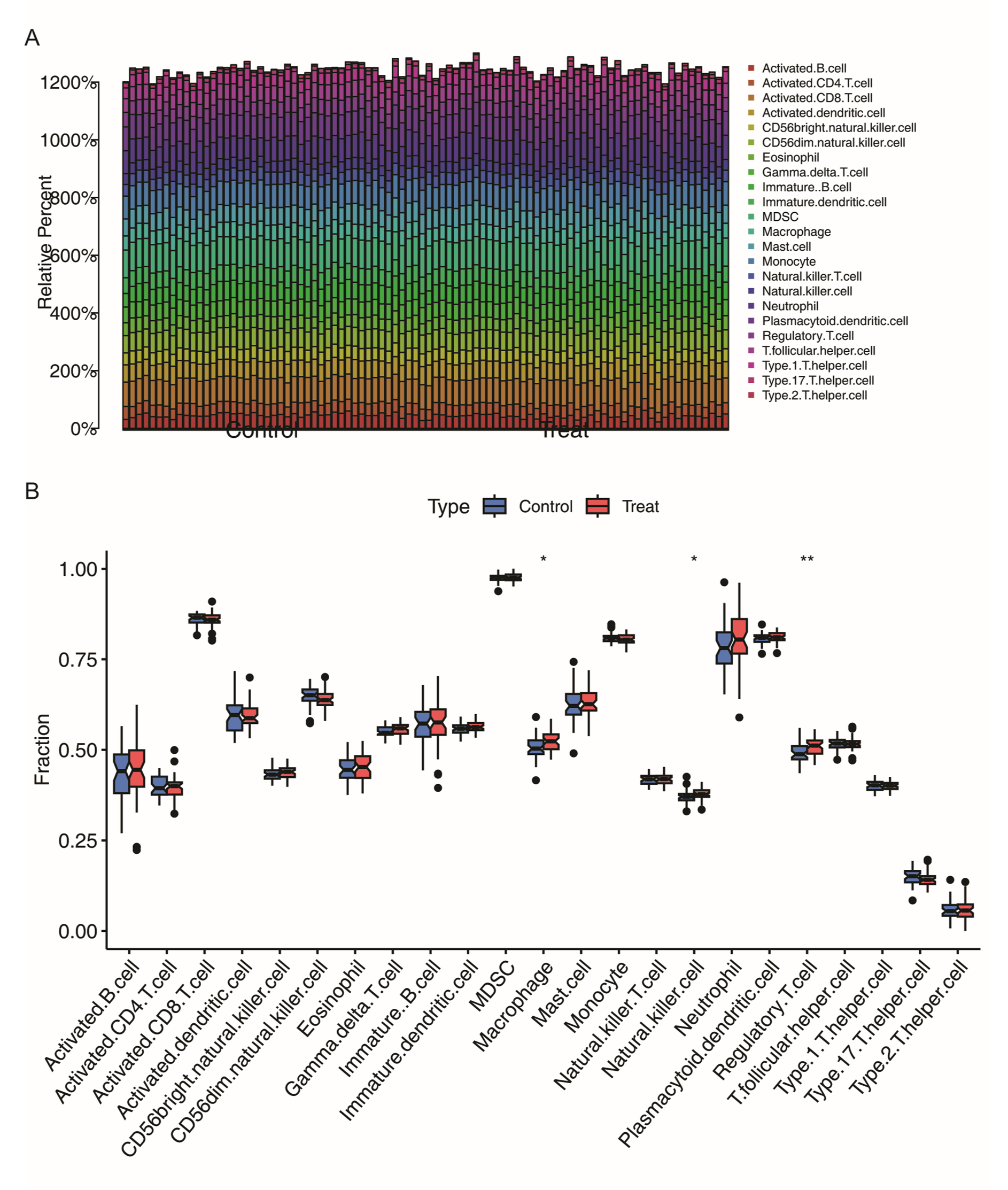

Supplement: Supplementary file 1 [file DataSheet1.zip › S1-S10/fig.S4.TIF]

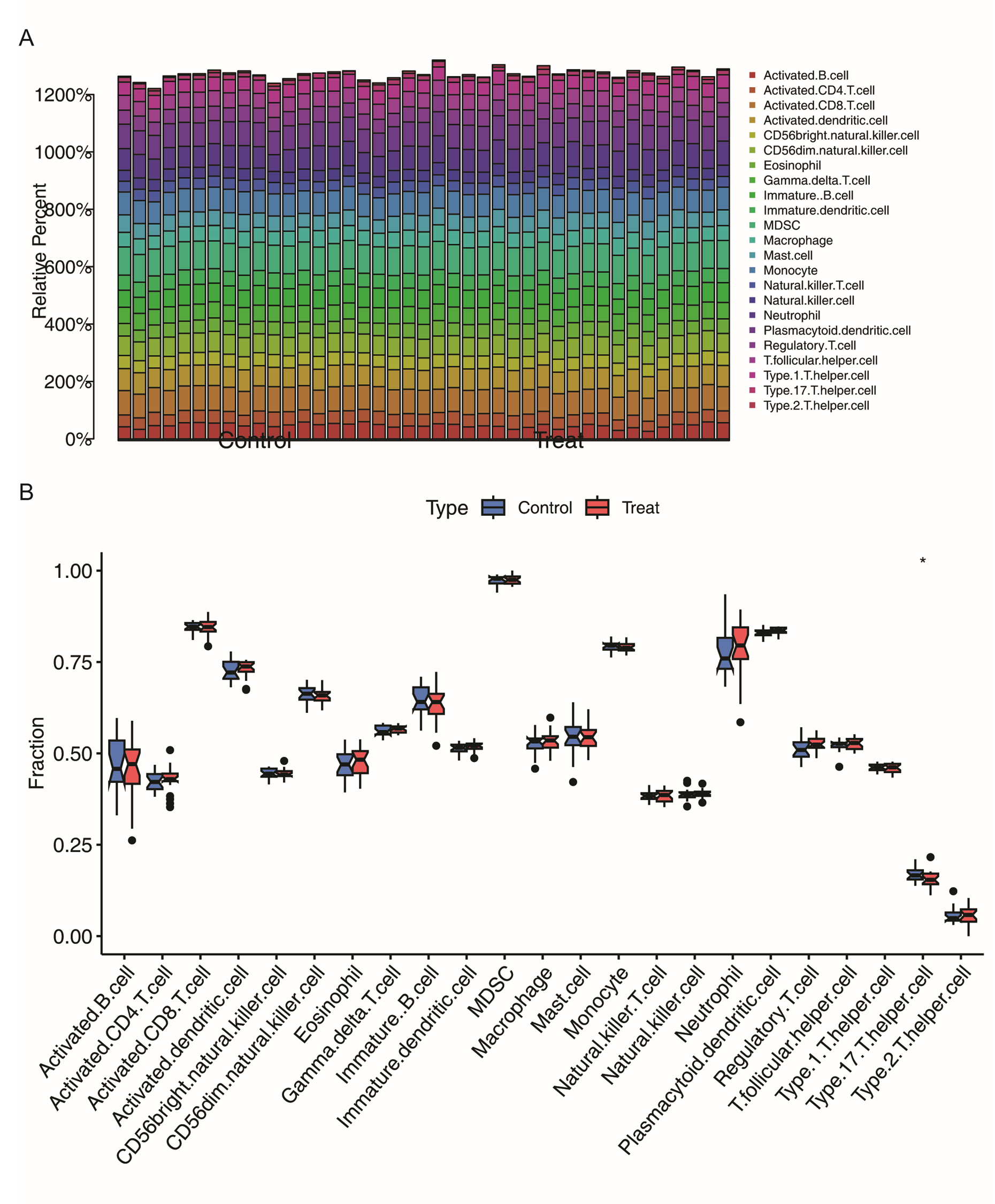

Supplement: Supplementary file 1 [file DataSheet1.zip › S1-S10/fig.S5.TIF]

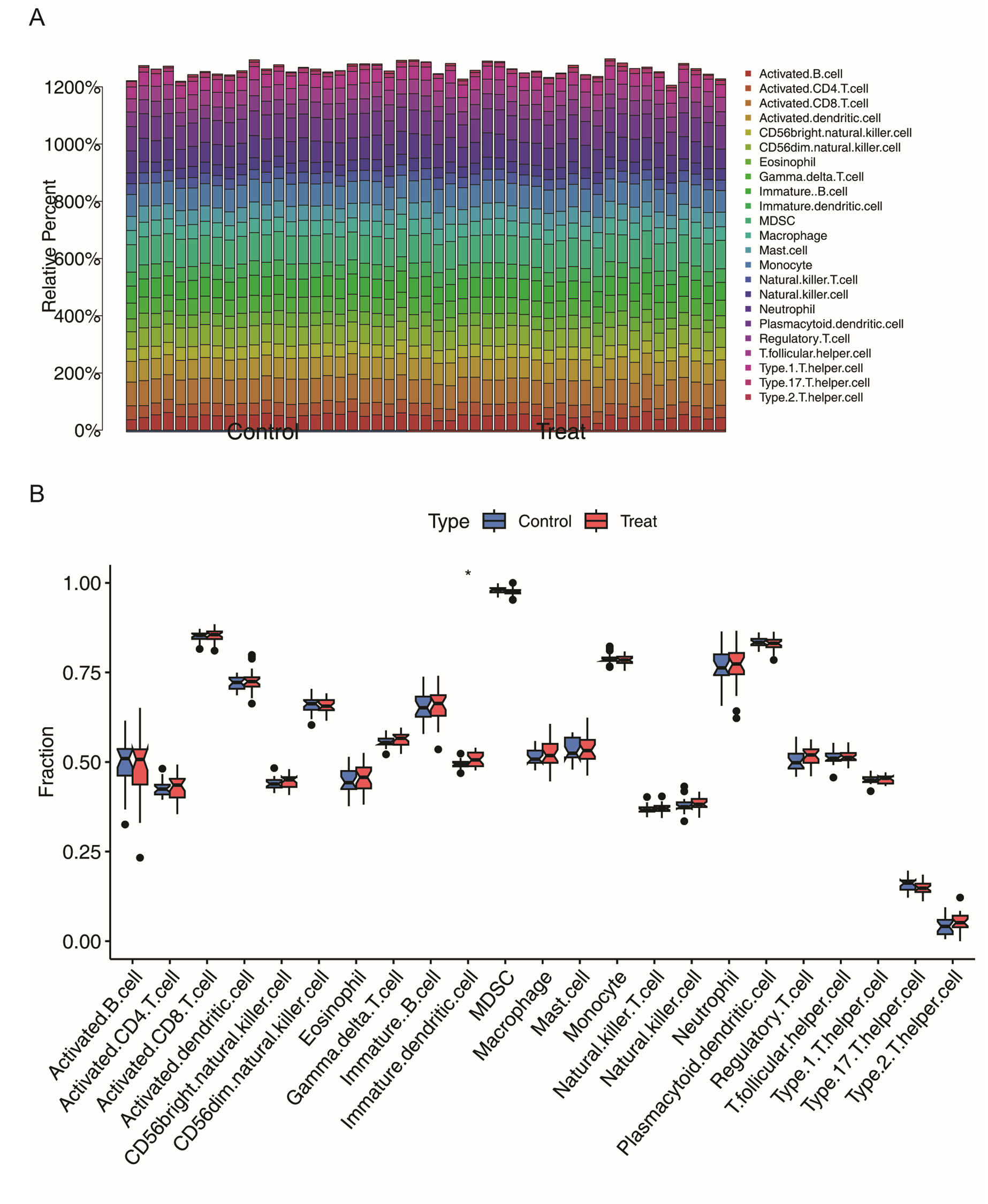

Supplement: Supplementary file 1 [file DataSheet1.zip › S1-S10/fig.S6.TIF]

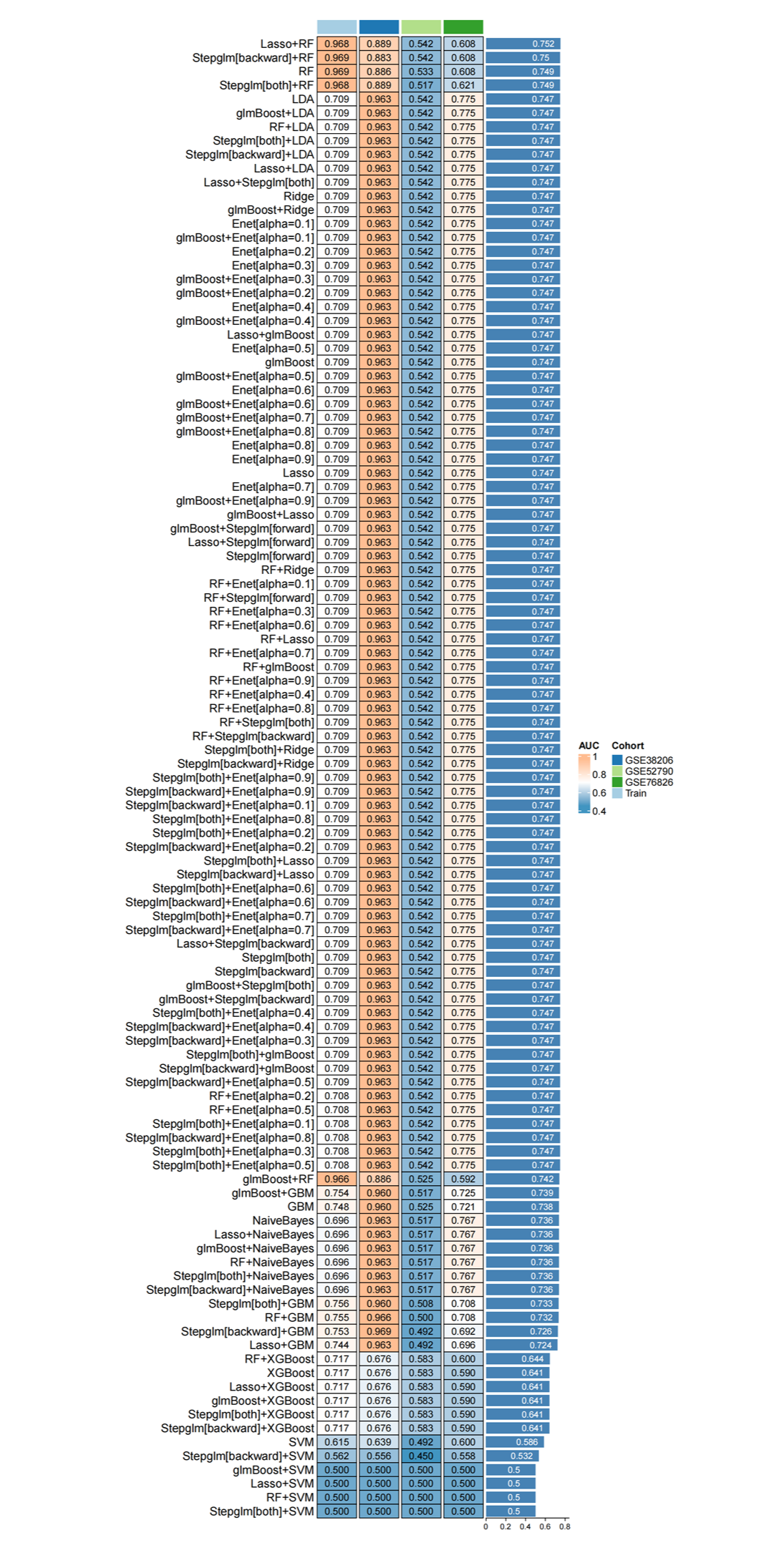

Supplement: Supplementary file 1 [file DataSheet1.zip › S1-S10/fig.S7.TIF]

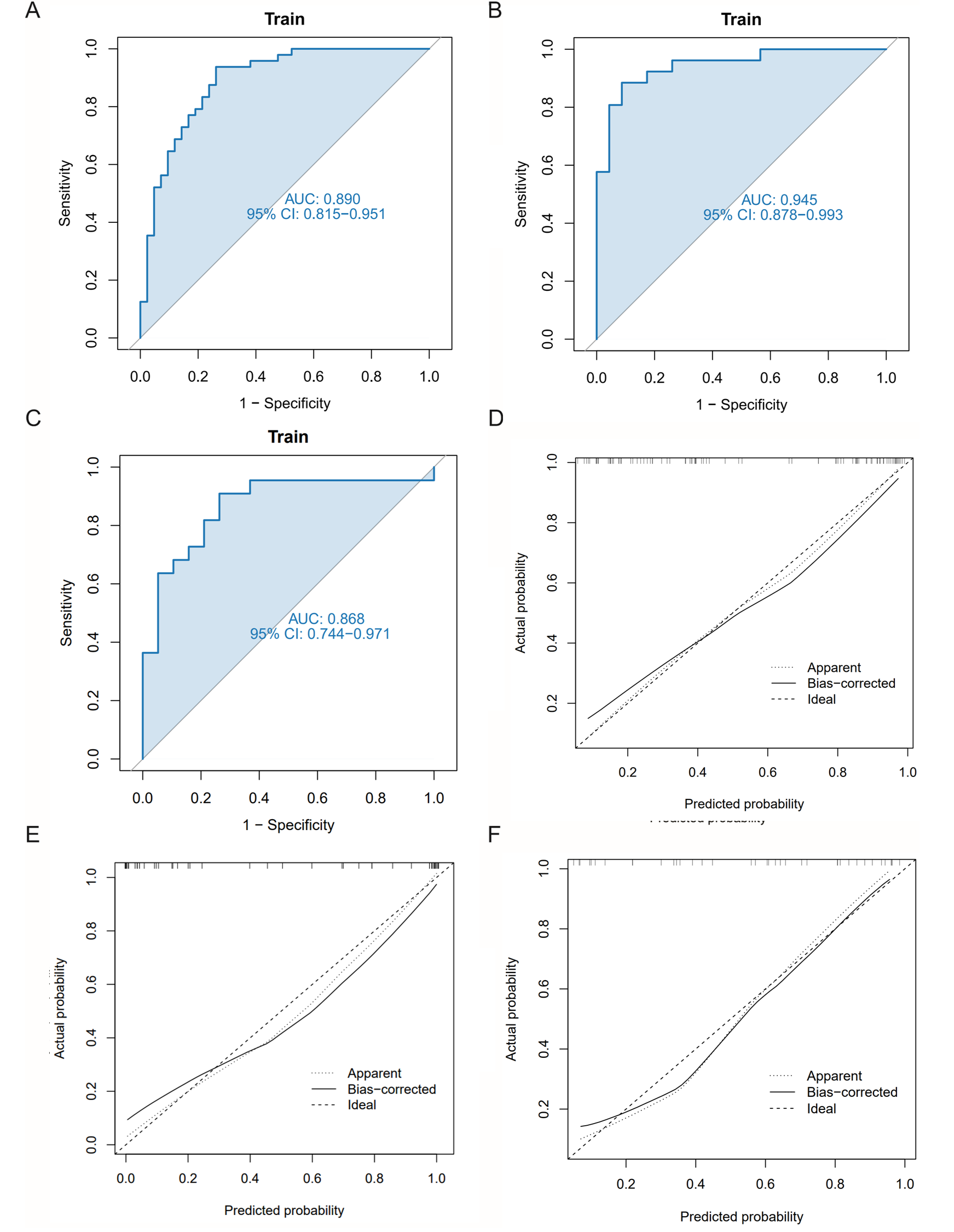

Supplement: Supplementary file 1 [file DataSheet1.zip › S1-S10/fig.S8.TIF]

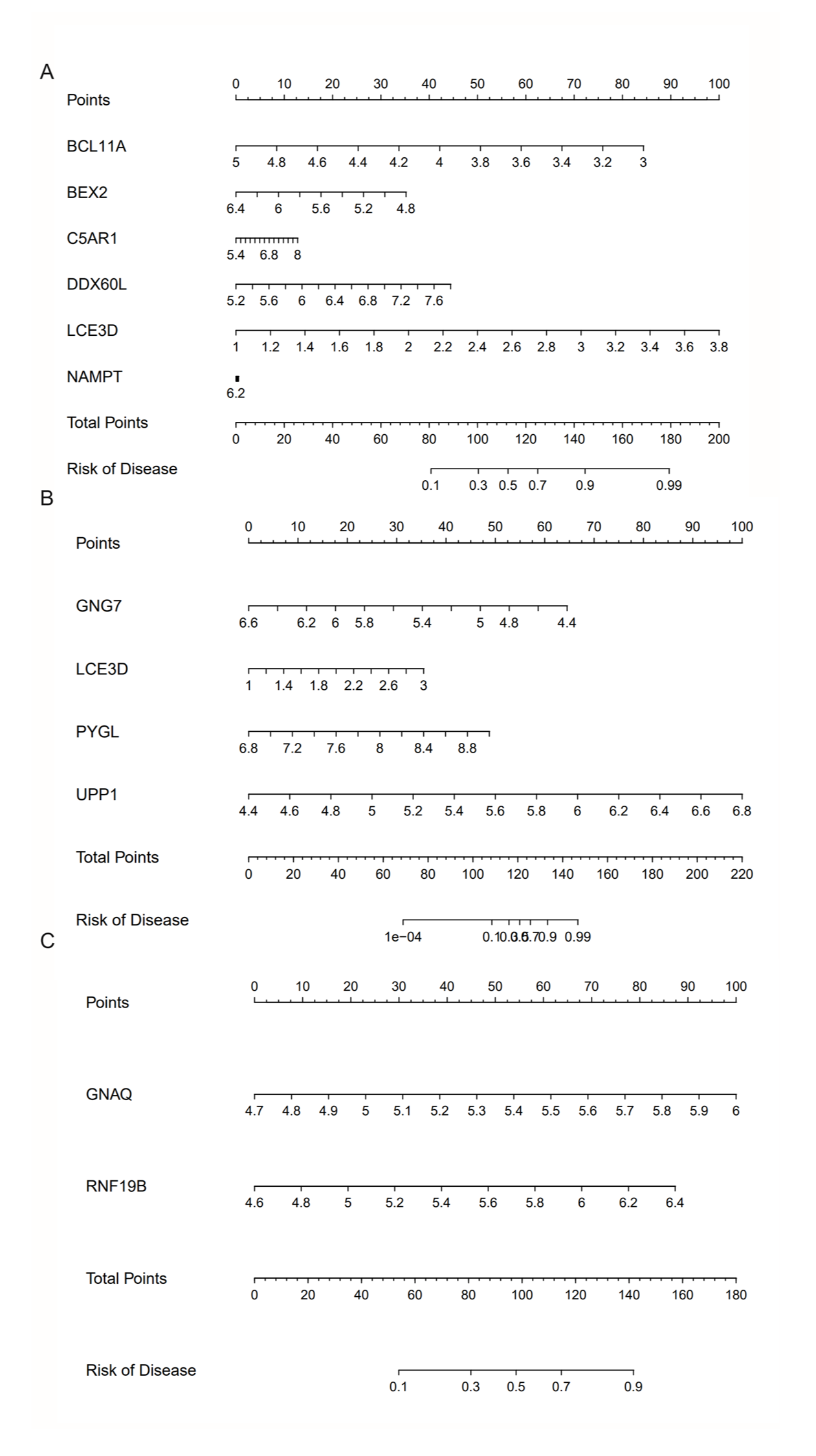

Supplement: Supplementary file 1 [file DataSheet1.zip › S1-S10/fig.S9.TIF]
