# Supplementary material for: Commonalities and differences in gene expression patterns in major depressive disorder and chronic spontaneous urticaria: implications for comorbidity
Source: Front Genet. 2025 Jul 29;16:1560832. doi: 10.3389/fgene.2025.1560832 (PMC12339353; doi:10.3389/fgene.2025.1560832)
Supplement: Supplementary file 2 [file DataSheet3.pdf]

Supplementary Material

1 SUPPLEMENTARY FIGURES

1.1 Figures

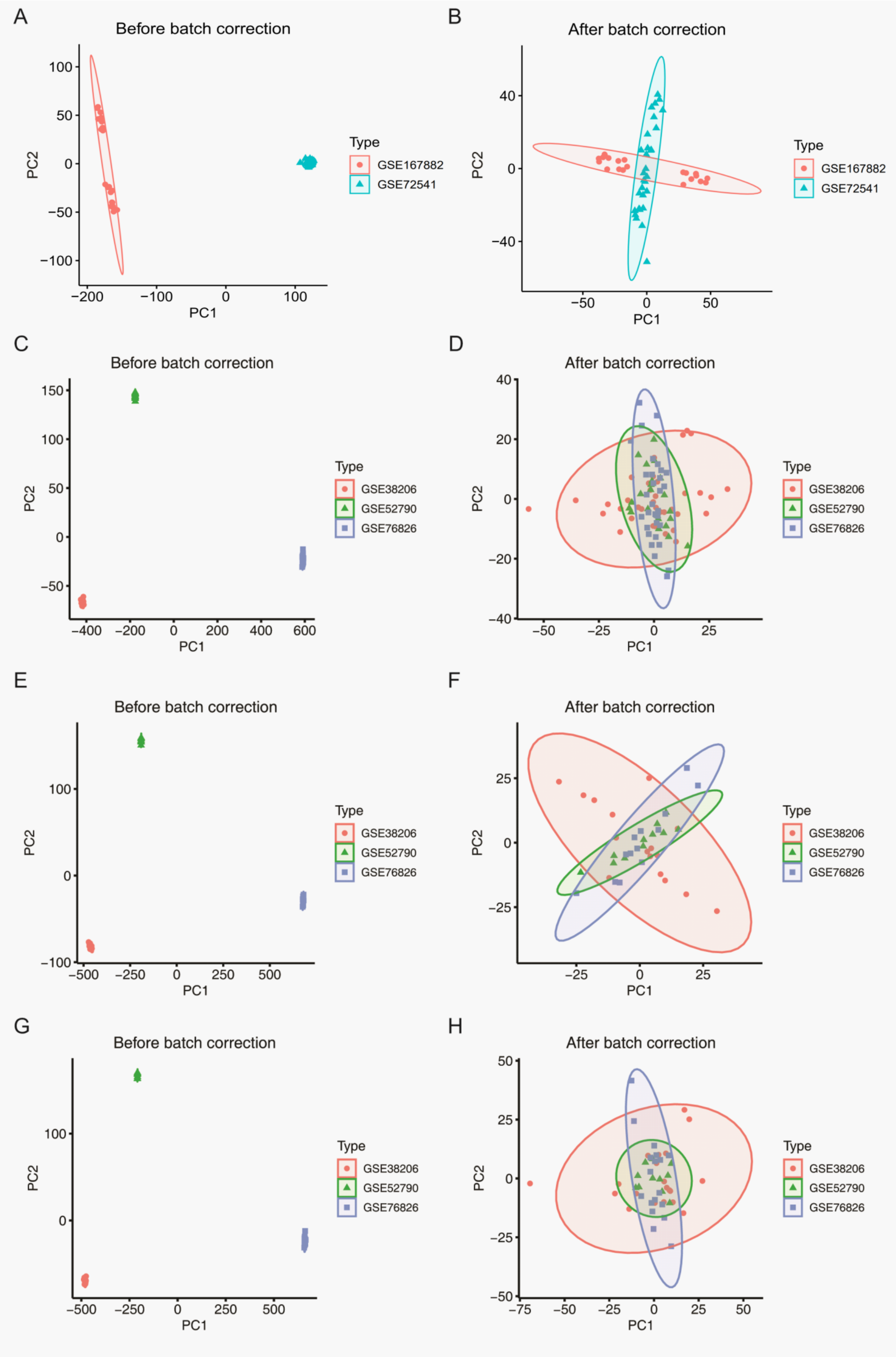

Figure S1. The integration of MDD datasets and CSU datasets. (A) PCA of three original CSU datasets prior to batch effect correction. (B) PCA of integrated CSU dataset after batch effect correction. (C) PCA of the total MDD population dataset before batch effect correction. (D) PCA of the total MDD population dataset following batch effect correction. (E) PCA of the male MDD dataset before batch effect correction. (F) PCA of the male MDD dataset following batch effect correction. (G) PCA of the female MDD dataset before batch effect correction. (H) PCA of the female MDD dataset following batch effect correction.

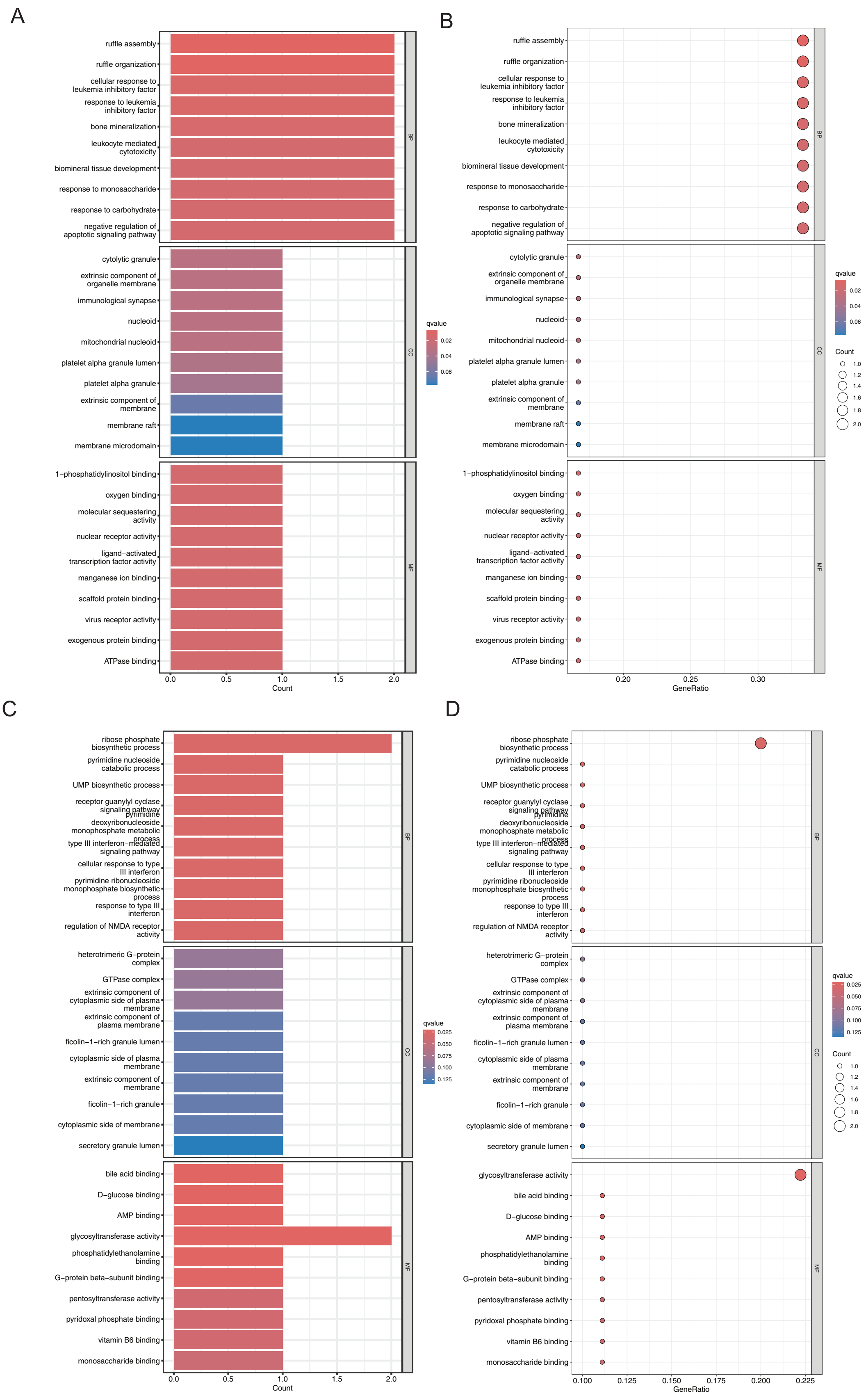

Figure S2. (A) Bar plot of GO enrichment analysis results in the male population in MDD. (B) Bubble plot of GO enrichment analysis results in the male group in MDD. (C) Bar plot of GO enrichment analysis results in the female group in MDD. (D) Bubble plot of GO enrichment analysis results in the female group in MDD.

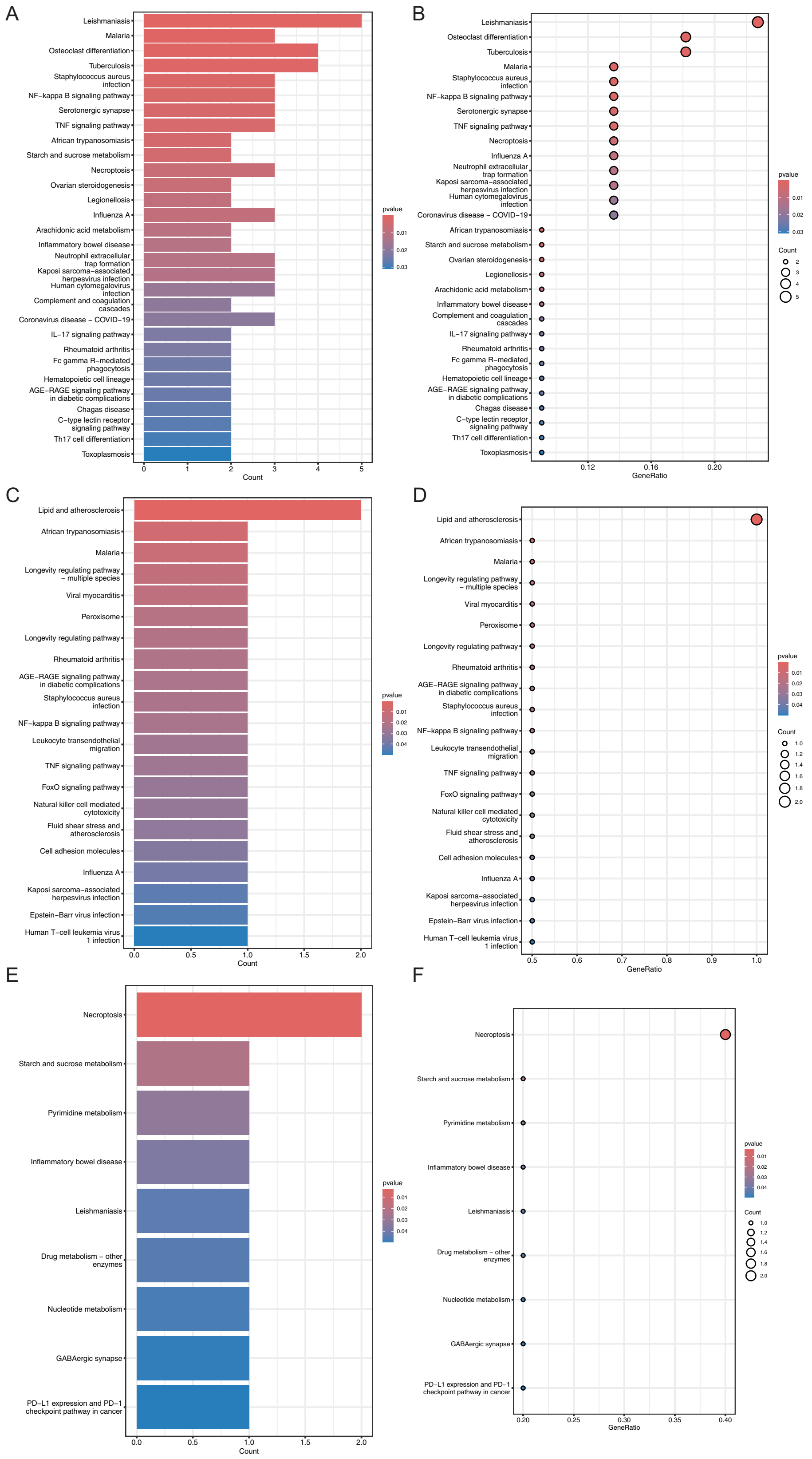

Figure S3. (A) Bar plot of KEGG pathway enrichment analysis for the total population. (B) Bubble plot of KEGG pathway enrichment analysis for the total group. (C) Bar plot of KEGG pathway enrichment analysis for the male group in MDD. (D) Bubble plot of KEGG pathway enrichment analysis for the male population in MDD. (E) Bar plot of KEGG pathway enrichment analysis for the female group in MDD. (F) Bubble plot of KEGG pathway enrichment analysis for the female group in MDD.

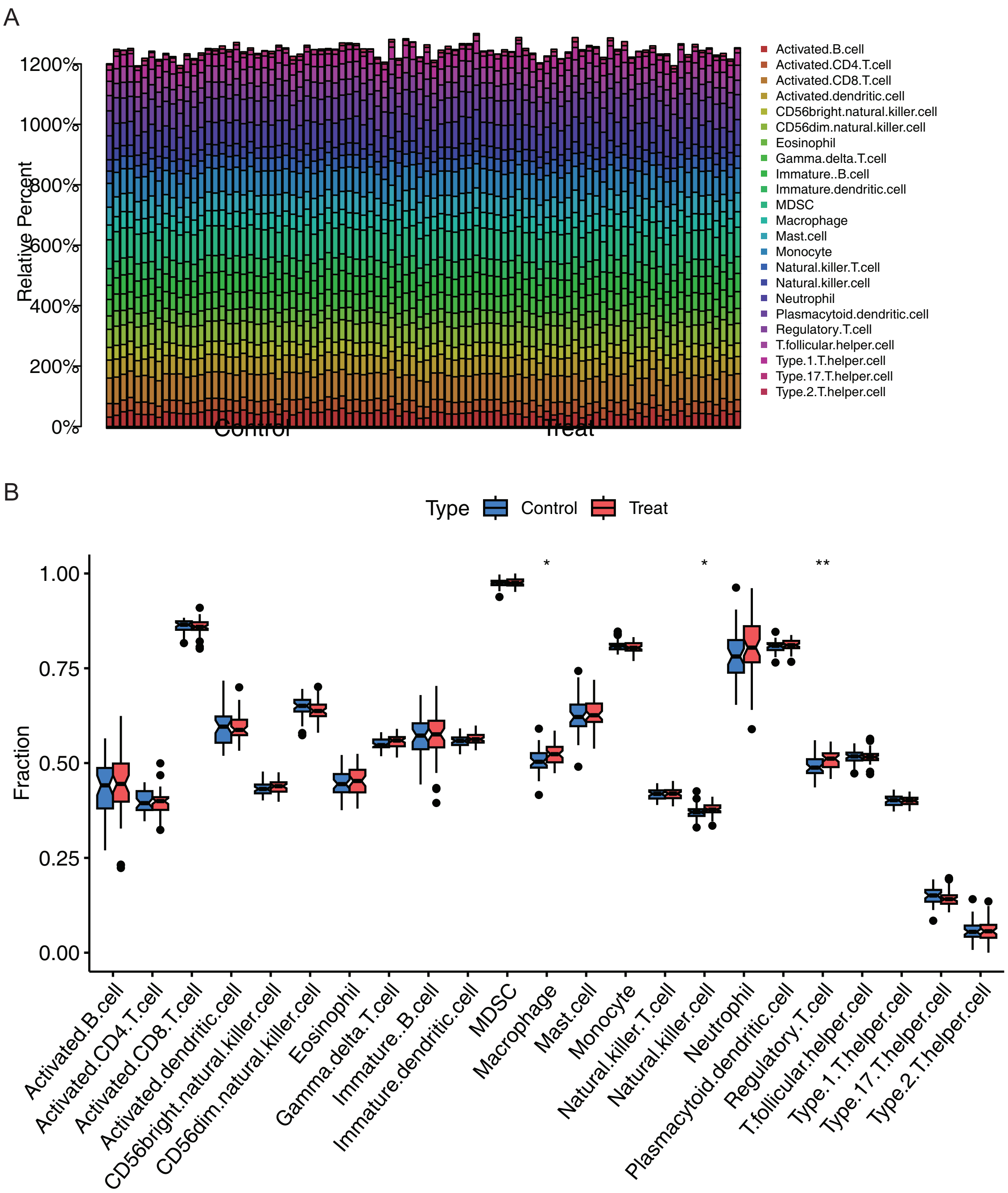

Figure S4. (A) Bar plot showing immune cell abundances in MDD patients and controls (total population). (B) Immune cell analysis (boxplots) comparing immune cell abundances in MDD patients and controls (total population).

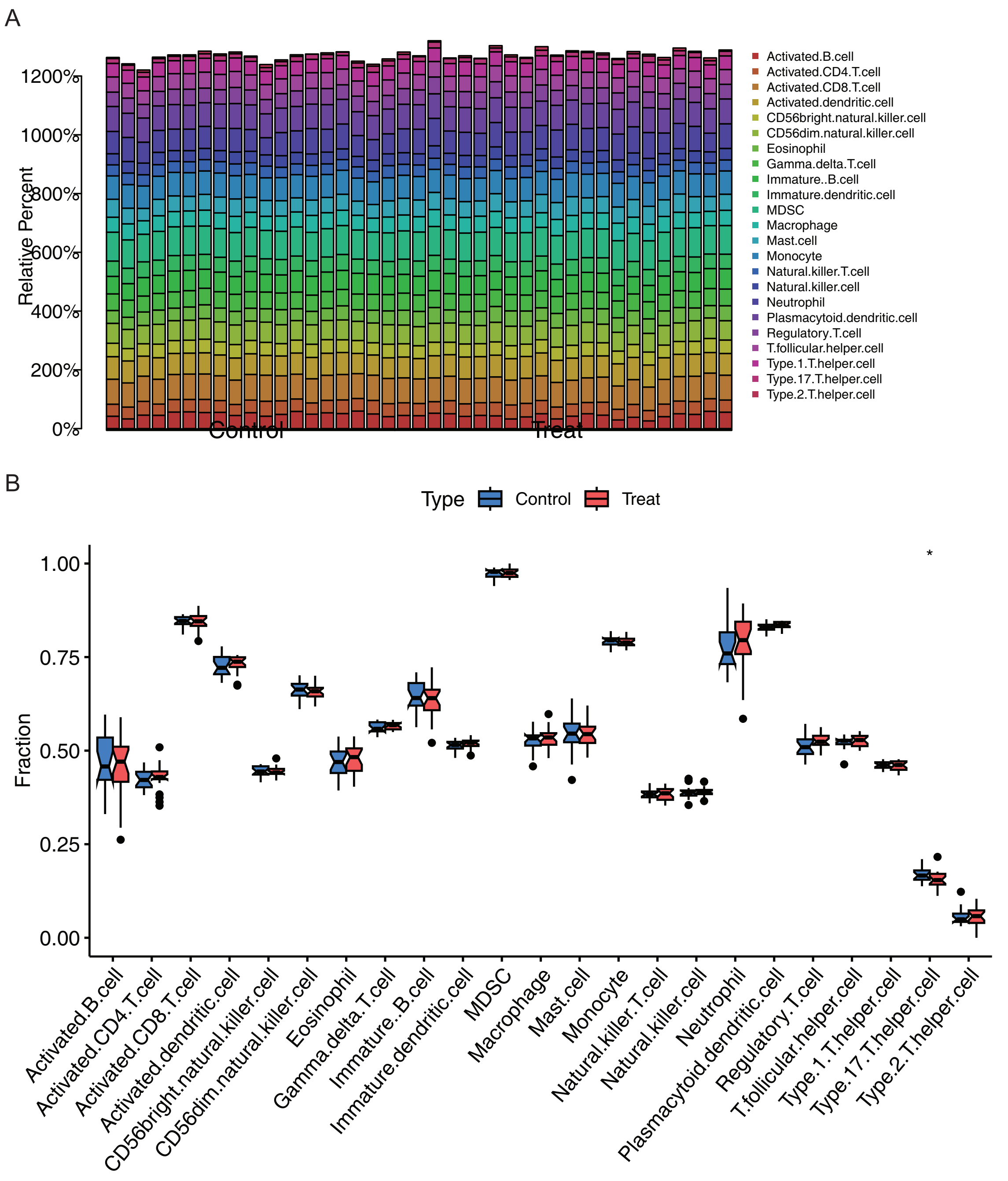

Figure S5. (A) Bar plot showing immune cell abundances in MDD male patients and controls. (B) Immune cell analysis (boxplots) comparing immune cell abundances in MDD male patients and controls.

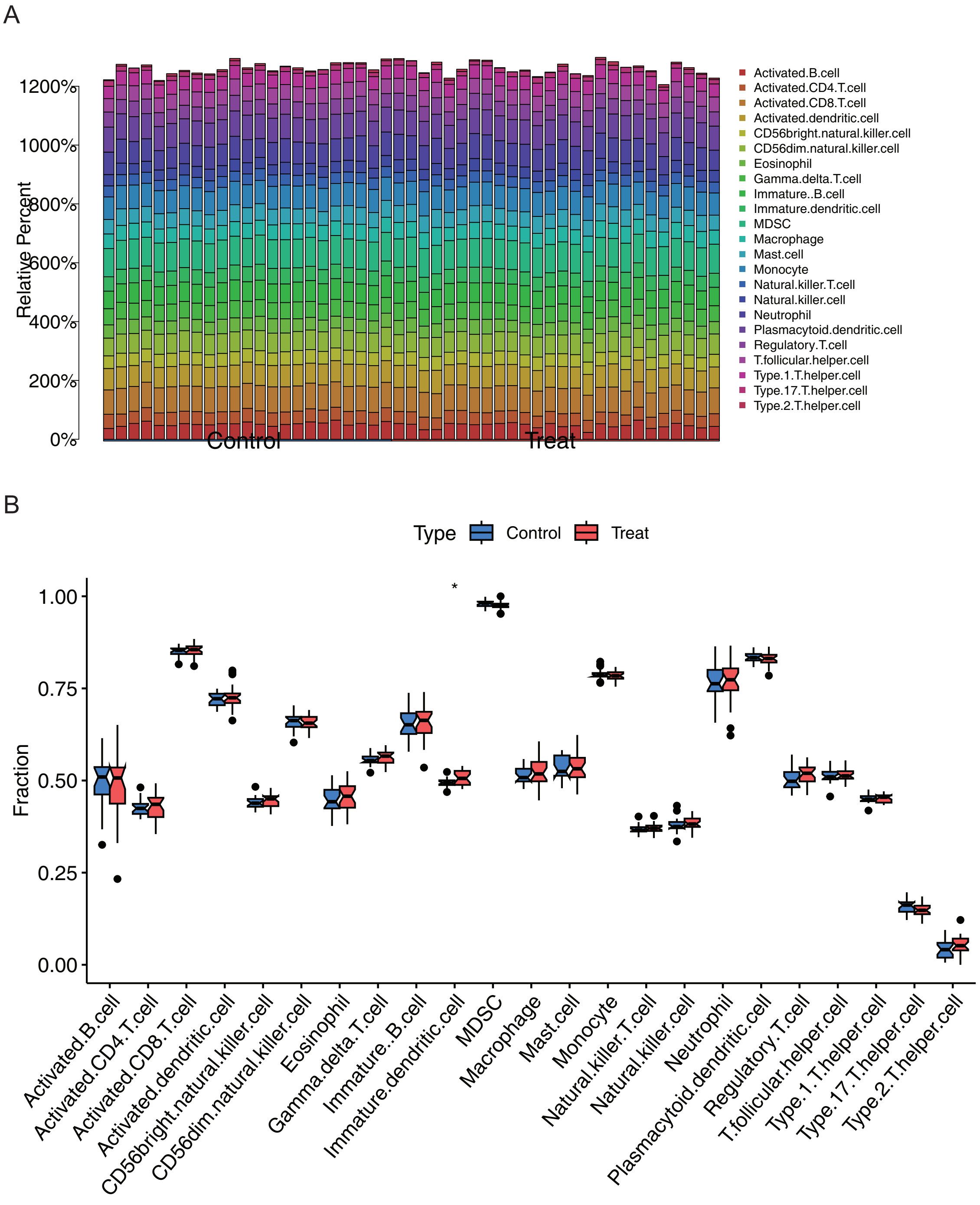

Figure S6. (A) Bar plot showing immune cell abundances in MDD female patients and controls. (B) Immune cell analysis (boxplots) comparing immune cell abundances in MDD female patients and controls.

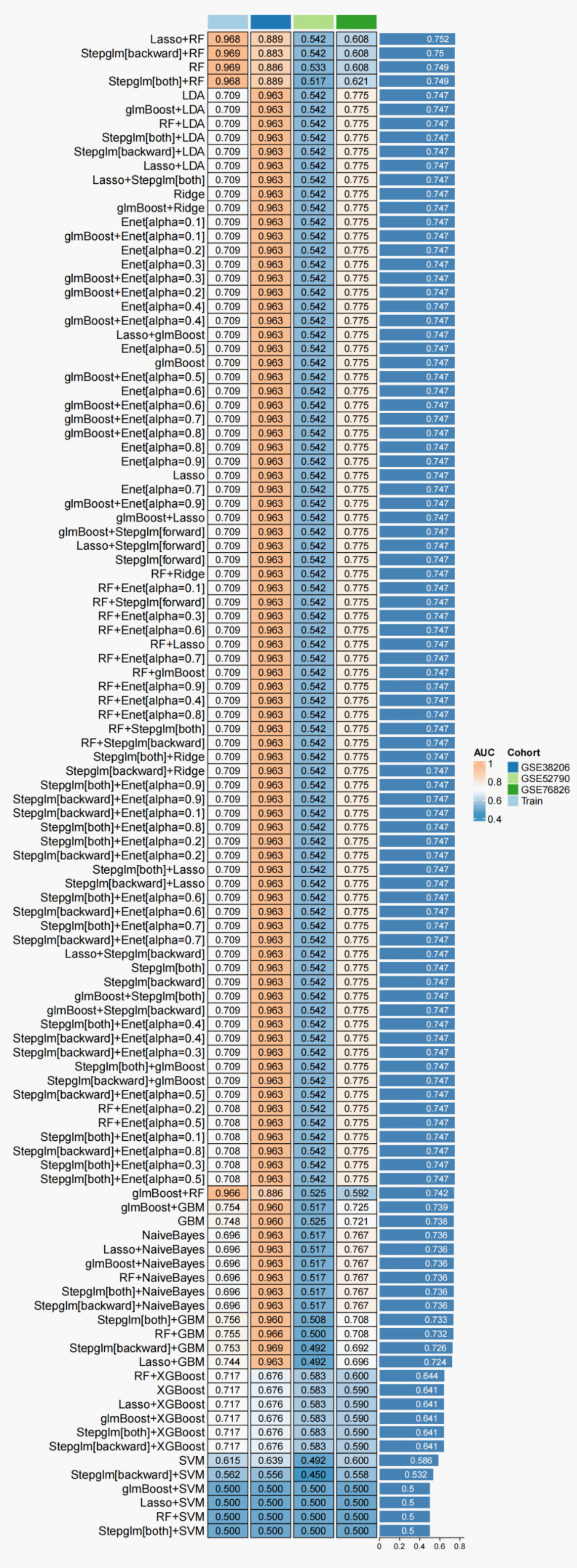

Figure S7. Machine learning transcriptomic classification model evaluated for the male group. ROC Curves:

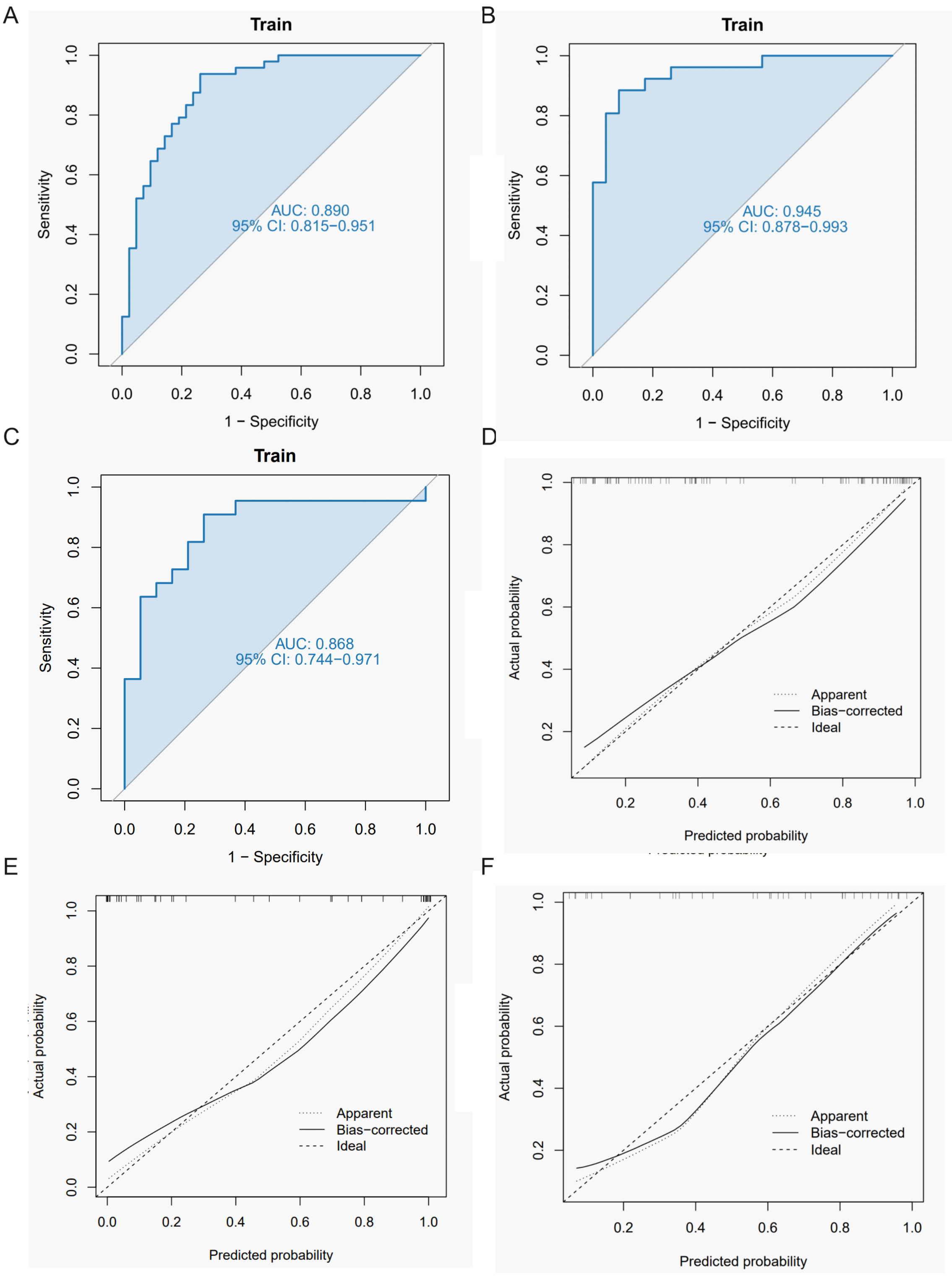

Figure S8. (A) ROC curve of the transcriptomic classification model for the total population. (B) ROC curve of the transcriptomic classification model for the male group. (C) ROC curve of the transcriptomic classification model for the female group. Calibration Curves, Nomograms, and DCA Analysis: (D) Calibration curve of the transcriptomic classification model for the total population. (E) Calibration curve of the transcriptomic classification model for the male group. (F) Calibration curve of the transcriptomic classification model for the female group.

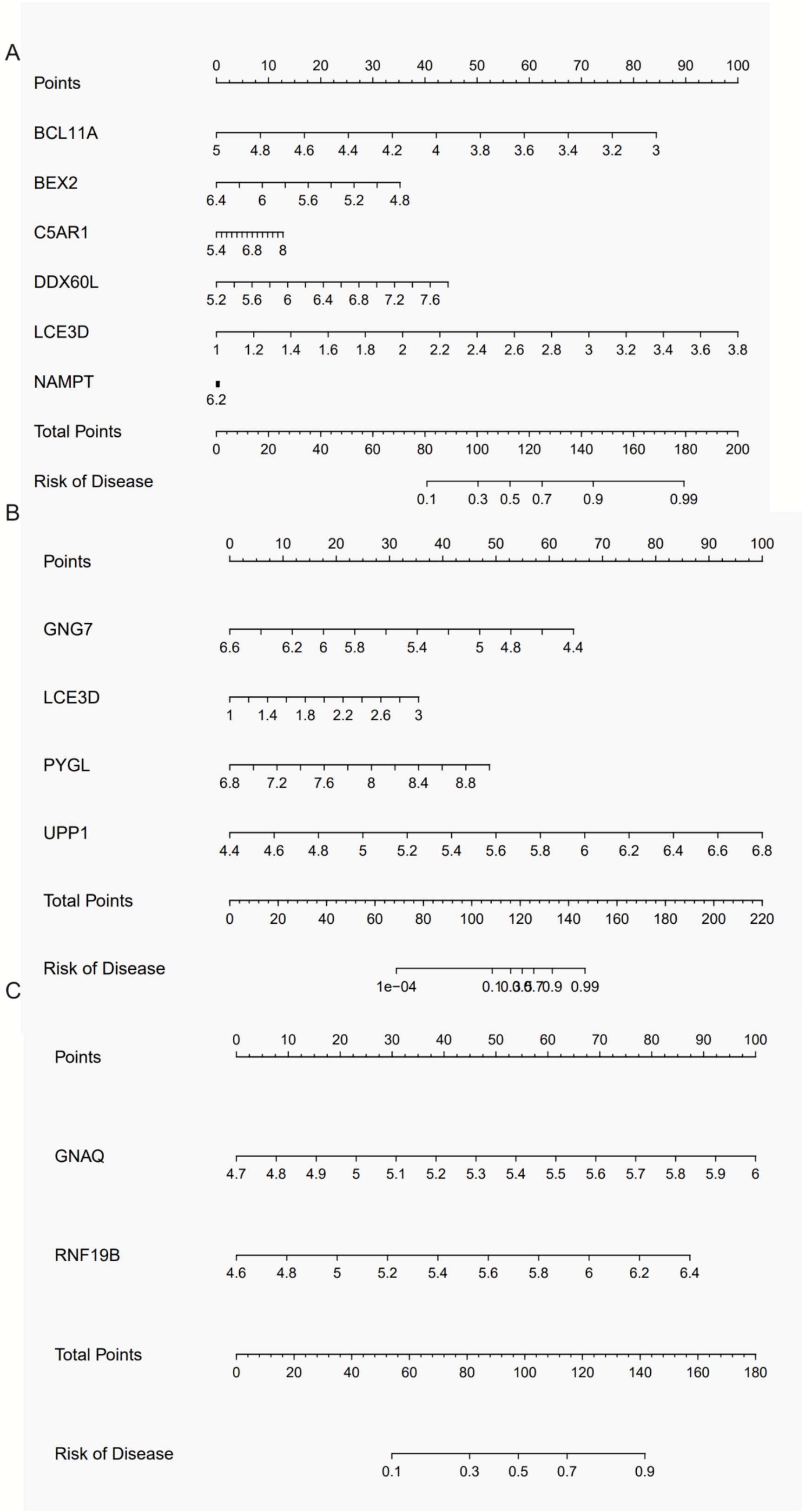

Figure S9. (A) Nomogram of the transcriptomic classification model for the total population. (B) Nomogram of the transcriptomic classification model for the male group. (C) Nomogram of the transcriptomic classification model for the female group.

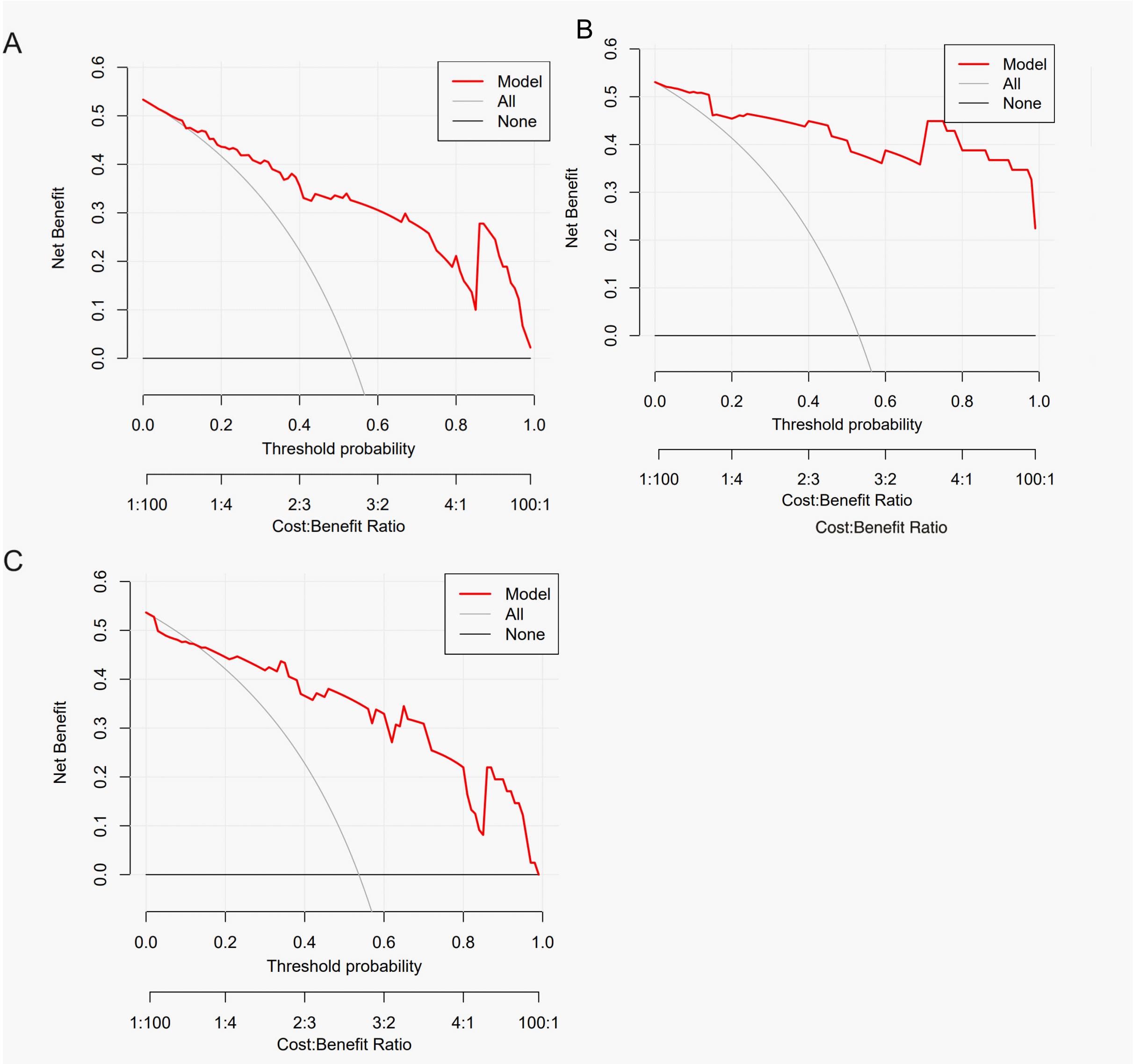

Figure S10. (A) Decision curve analysis (DCA) of the transcriptomic classification model for the total population. (B) Decision curve analysis (DCA) of the transcriptomic classification model for the male group. (C) Decision curve analysis (DCA) of the transcriptomic classification model for the female group.
